# Targeted quantitative metabolomics with a linear mixed-effect model for analysis of urinary nucleosides and deoxynucleosides from bladder cancer patients before and after tumor resection

**DOI:** 10.1007/s00216-023-04826-0

**Published:** 2023-07-18

**Authors:** Małgorzata Artymowicz, Wiktoria Struck-Lewicka, Paweł Wiczling, Marcin Markuszewski, Michał J. Markuszewski, Danuta Siluk

**Affiliations:** 1 Department of Biopharmaceutics and Pharmacodynamics, Medical University of Gdańsk, Aleja Gen. J. Hallera 107, 80-416, Gdańsk, Poland; 2 Department of Urology, Medical University of Gdańsk, Mariana Smoluchowskiego 17, 80-214 Gdańsk, Poland

**Keywords:** Modified nucleosides, Modified deoxynucleosides, Targeted metabolomics, Method validation, Bladder cancer, Linear mixed-effect model

## Abstract

In the present study, we developed and validated a fast, simple, and sensitive quantitative method for the simultaneous determination of eleven nucleosides and deoxynucleosides from urine samples. The analyses were performed with the use of liquid chromatography coupled with triple quadrupole mass spectrometry. The sample pretreatment procedure was limited to centrifugation, vortex mixing of urine samples with a methanol/water solution (1:1, *v/v*), evaporation and dissolution steps. The analysis lasted 20 min and was performed in dynamic multiple reaction monitoring mode (dMRM) in positive polarity. Process validation was conducted to determine the linearity, precision, accuracy, limit of quantification, stability, recovery and matrix effect. All validation procedures were carried out in accordance with current FDA and EMA regulations. The validated method was applied for the analysis of 133 urine samples derived from bladder cancer patients before tumor resection and 24 h, 2 weeks, and 3, 6, 9, and 12 months after the surgery. The obtained data sets were analyzed using a linear mixed-effect model. The analysis revealed that concentration level of 2-methylthioadenosine was decreased, while for inosine, it was increased 24 h after tumor resection in comparison to the preoperative state. The presented quantitative longitudinal study of urine nucleosides and deoxynucleosides before and up to 12 months after bladder tumor resection brings additional prospective insight into the metabolite excretion pattern in bladder cancer disease. Moreover, incurred sample reanalysis was performed proving the robustness and repeatability of the developed targeted method.

**Graphical abstract:**

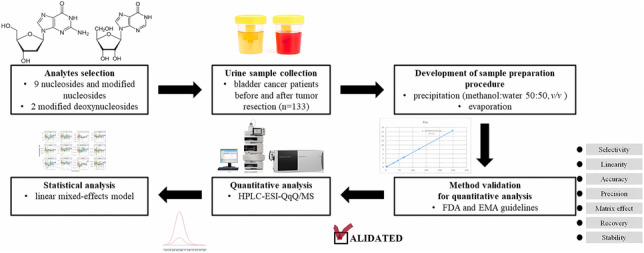

**Supplementary Information:**

The online version contains supplementary material available at 10.1007/s00216-023-04826-0.

## Introduction

Concentrations of exogenous and endogenous metabolites detected in biological matrices are often altered depending on the physiological human body condition or its pathophysiological state. Thanks to the development of advanced analytical techniques that can detect numerous heterogenous compounds, commonly used laboratory tests can quickly and reliably determine their exact concentrations even at trace levels. Nevertheless, there is still a need to develop and implement analytical procedures for new sets of compounds whose presence or altered concentrations would indicate the clinical health status. Such efforts are carried out mainly in the field of targeted metabolomics studies, where an exact group of various metabolites, often selected on the basis of untargeted metabolomics analysis, are quantitatively determined along with the assessment of their potential clinical importance in such severe diseases as cancer, cardiovascular disease, neurodegenerative disorders, and others [1, 2].

In regard to cancer-related metabolites, nucleosides and deoxynucleosides might play a crucial role as potential biomarkers of disease progress or remission [3–5]. Nucleosides and deoxynucleosides are the end products of the degradation of ribo- (RNA) and deoxyribonucleic acid (DNA), respectively. Both these groups of metabolites are low molecular weight compounds composed of purine or pyrimidine linked to ribose in the case of nucleosides or to deoxyribose in the case of deoxynucleosides. It is documented that RNA turnover processes are faster in such conditions as intensive cell growth, inflammation, hemopathies, and malignant diseases [3]. In such disorders, nucleosides (adenosine, guanosine, uridine, cytidine) are further metabolized to uric acid, β-alanine, and β-aminoisobutyrate or they can be used for rebuilding RNA. Apart from nucleosides, their modified analogues (as methylated or acetylated forms) are excreted intact into urine. Since the correlation of elevated levels of nucleosides and their modified analogues with faster RNA turnover in the case of malignant diseases has been documented, these metabolites have been examined in terms of their usefulness as potential cancer biomarkers [6–10]. Likewise, deoxynucleosides, compounds that are final products of oxidative DNA damage, often occur during oxidative stress processes as well as during other epigenetic DNA modifications. The oxidative stress can also trigger numerous diseases such as cancer, cardiovascular diseases, or neurodegenerative disorders [11–14]. Therefore, nucleosides along with deoxynucleosides can be determined in biological fluids for the sake of their potential clinical importance in tumorigenesis [15–20].

Bladder cancer (BCa) is the tenth most common diagnosed type of cancer worldwide [21]. It is four times more common in men than women, and the risk of the disease increases with age. The main risk factors for occurring bladder cancer are genetic susceptibility, tobacco smoking, and occupational exposure to carcinogens such as benzidine, 4-aminobiphenyl, 2-naphthylamine, and 4-chloro-o-toluidine [21–23]. The most common symptom of bladder cancer is macroscopic or microscopic visible blood in urine (hematuria). Macroscopic hematuria is considered as symptom of advanced stage of disease. In patients with microscopic hematuria, bladder cancer is often discovered incidentally during routine medical examinations. Screening tests for bladder cancer are not available. Bladder cancer usually develops from the epithelium (urothelium) that covers the inner surface of the bladder. If the tumor invades the muscles of the bladder, it is called muscle-invasive bladder cancer (MIBC). This type of cancer is less common, but more likely to metastasize to lymph nodes and other organs. However, 75% of all cases of bladder cancer account for non-invasive bladder cancer (NMIBC). It has a higher survival rate but can recur frequently and progress to MIBC. The standard in the diagnosis and treatment of NMIBC is the transurethral resection of bladder tumor surgery (TURBT). Due to the risk of recurrence, patients after TURBT have to undergo frequent doctor visits and cystoscopy examinations, which in total generates high costs of bladder cancer treatment.

The metabolomics approach has been used several times in bladder cancer research [24–31]. The main goal of the metabolomics studies was to identify metabolites that differ in the compared groups (usually patients diagnosed with bladder cancer vs. healthy volunteers), so as to potentially enrich the knowledge about bladder cancer. Metabolomics analyses mainly focused on untargeted analyses and utilized urine samples [25, 27–30] as well as bladder tissue [26, 30, 31] and plasma [24]. Those studies focused only on patients diagnosed with muscle-invasive bladder cancer [25] or the study group consisted of patients with various types of bladder cancer. Various analytical techniques were used and metabolomics analyses were combined with transcriptomic and lipidomic analyses [26, 31]. Accordingly, a number of different compounds have been identified as differentiating controls from bladder cancer patients. Two studies indicate uridine as a statistically significant metabolite [25, 26], and one study indicates 8-hydroxy-deoxyguanosine [26]. 8-Hydroxy-deoxyguanosine has previously been studied in patients diagnosed with bladder cancer as a potential indicator of the disease [32, 33].

Based on the results of our earlier studies, 8 nucleosides and modified nucleosides were selected to be analyzed in the present study, namely, pseudouridine, uridine, N3-methyluridine, inosine, N6-methyladenosine, N2-methylguanosine, N2,N2-dimethylguanosine, and 5-methylthioadenosine [8, 16, 34–37]. It should also be emphasized that previously conducted research was focused on the role of nucleosides and their modified analogues, while modified deoxynucleosides were not included in the studies. There was also no method developed for their simultaneous extraction with modified nucleosides from urine samples. Moreover, up to now, not many works are available that present the simultaneous determination of nucleosides and deoxynucleosides in biological samples [34, 38]. Unfortunately, these compounds differ in their physicochemical properties. Well-known and widely used extraction methods in the analysis of nucleosides and their modified analogues based on phenyl-boronate sorbents cannot be used in the case of deoxynucleosides due to the lack of cis-diol bonds. However, due to the fact that both modified nucleosides and deoxynucleosides have been indicated as potential biomarkers of various pathological conditions, it is advisable to develop a method enabling their simultaneous analysis in biological samples. Because of that, based on the literature research, three metabolites were also included in the study, namely, 2-methyltioadenosine and two most frequently mentioned as significant deoxynucleosides: 2-deoxyguanosine and 8-hydroxy-2-deoxyguanosine [3, 11, 12, 14]. Our previously quantitative analyses included comparison of two groups—patients vs healthy volunteers. Generally, so far, numerous studies have been focused on nucleoside determination only at one time point (healthy vs control group) [28, 29, 35–37] or from postoperative bladder cancer patients with or without recurrence [39] or at various stages of cancer state (benign bladder cancer vs advanced state) [30, 40]. Moreover, in our two previously performed projects, the study group consisted of patients with various urogenital cancers, including bladder cancer [8, 36]. As a continuation, it was decided to focus on one selected disease and to conduct a prospective analysis to assess how the concentrations of previously considered as statistically significant nucleosides and deoxynucleosides change in the situation of tumor resection and in a period of time after surgery.

Our aim was to develop and validate a fast and easy targeted quantitative metabolomics method for the simultaneous determination of 3 nucleosides along with their 6 modified analogues, and 2 deoxynucleosides, covering different time points before and up to 12 months after bladder tumor resection. The proposed method was validated according to the Food and Drug Administration (FDA) and the European Medicines Agency (EMA) criteria in terms of linearity, accuracy, precision, matrix effect, stability, limit of quantification, and selectivity [41, 42]. Next, the validated method was implemented for the analysis of 133 urine samples derived from bladder cancer patients before tumor resection and 24 h, 2 weeks, and 3, 6, 9, and 12 months after the surgery. Analysis was performed with the use of high-performance liquid chromatography hyphenated with triple quadrupole mass spectrometry (HPLC-QqQ/MS). Furthermore, a linear mixed-effects model (LMM) has been utilized to analyze the obtained longitudinal data. The LMM is considered to be robust for the analysis of data with complex, correlated structures and multiple sources of variation [43–45]. Taking into account the time course data which were obtained in this study, the LMM approach can identify possible differences over time, between patients, and/or within patient groups. The goal of data analysis was to verify whether changes in nucleoside and deoxynucleoside concentrations in urine over time are correlated with the resection of bladder tumor. To evaluate the robustness of the method over a long period of time, incurred sample reanalysis (ISR) was conducted.

## Materials and methods

### Materials and chemicals

Uridine (U), pseudouridine (Pse), N3-methyluridine (3mU), N2,N2-dimethylguanosine (2,2dmG), 2-methylthioadenosine (MTA), 8-bromoguanosine (8BrG, internal standard, IS), N2-methylguanosine (2mG), N6-methyladenosine (6mA), and 8-hydroxy-2-deoxyguanosine (8OH2dG) were obtained from Carbosynth (Berkshire, Great Britain). 2-deoxyguanosine (2dG), 5-methylthioadenosine (5-MTA), inosine (Ino), 0.1 N sodium hydroxide (NaOH), and 98–100% LC-MS-grade formic acid were purchased from Sigma-Aldrich (Schnelldorg, Germany). MS-grade methanol was purchased from Thermo Fisher Scientific (Waltham, MA, USA). Deionized water was obtained using Milli Ro and Milli-Q Plus apparatus (Millipore, Vienna, Austria). Synthetic urine (Surine® negative urine control), obtained from Supelco (Merck, Darmstadt, Germany), was used as a urine control.

### Apparatus

The method validation and its application to urine samples were covered with the use of an Agilent 1260 series high-performance liquid chromatography (HPLC) system coupled with a 6430 series triple quadrupole mass spectrometer (Agilent Technologies Inc., Santa Clara, CA, USA) with an electrospray ionization (ESI) source operated in positive polarity mode. The solution pH was adjusted with a Seven Compact TMS220 pH meter (Mettler-Toledo, Schwarzenbach, Switzerland). During sample preparation, the samples were vortex-mixed on an MS 3 Basic device (IKA, USA) and evaporated to dryness with the use of a GeneVac vacuum centrifuge (MiVac DUO Concentrator, Great Britain).

### Preparation of stock and working solutions

Standard stock solutions were prepared separately for each analyte by dissolving in a methanol:water solution (1:1, *v/v*), except for 2,2dmG and 2mG, which were dissolved in an aqueous solution of 1 M NaOH. Final concentrations of stock solutions were as follows: 10 mM for Pse and 2,2dmG; 2 mM for 2mG; 1 mM for U, I, 3 mU, and 5-MTA; 100 µM for 2dG, 8OH2dG, and 6 mA; and 10 µM for MTA. Stock solutions were stored at − 80 °C. In order to assure accurate analyte determination, two mixtures of working solutions were prepared. The first working solution was prepared by mixing the proper amounts of each stock solution with methanol to the final volume of 1 mL. The second working solution was prepared by the addition of 100 µL of the first working solution to 900 µL of methanol. Working standard solutions were kept at  − 20 °C. The proper volume of each working solution was added to the matrix in order to prepare quality control (QC) samples and calibration standards during the validation process.

### Urine collection

Fifty-three patients diagnosed with bladder cancer, treated at the Department of Urology, Medical University of Gdańsk (Gdańsk, Poland), were enrolled in the study. Informed consent, signed by each participant, was obtained before the collection of biological samples. Urine samples were collected at 7 time points: 24 h before the TURBT (*n* = 53) surgery and then 24 h (*n* = 50), 2 weeks (*n* = 10), and 3 (*n* = 8), 6 (*n* = 7), 9 (*n* = 3), and 12 (*n* = 2) months after the operation. The collected samples were kept frozen at − 80 °C until analysis. The presented project was approved by the Independent Committee of Bioethical Research at the Medical University of Gdańsk (Gdańsk, Poland) (number of consent: NKBBN/644/2018). The characteristics of patients enrolled in the study are presented in supplementary materials as Table SM1.

### Preparation of urine and blank urine samples

For validation purposes, blank urine samples were spiked with the proper amount of the working solutions and prepared in the same manner as the urine samples obtained from patients. One hundred microliters of centrifuged urine samples (14,800 × g, 10 min) was mixed with 10 µL of the internal standard and 900 µL of methanol:water (50:50, *v/v*). The mixture was vortex-mixed for 5 min. The samples were evaporated to dryness (45 °C, 1.5 h), and the dry residues were dissolved in 100 µL of methanol:water (1:1, *v/v*) solution and vortex-mixed for 5 min. The samples were transferred to inserts and the inserts were centrifuged (14,800 × g, 10 min).

### HPLC-QqQ/MS analysis

Chromatographic separation was achieved using a Zorbax Sb-Aq column from Agilent Technologies (3.0 × 100 mm, particle size 3.5 µm) under gradient conditions using 0.1% formic acid in water (mobile phase A) and 0.1% formic acid in methanol (mobile phase B) as the mobile phase mixture. The gradient started from 1 to 17% of B from 0 till 13 min, then increased to 25% of B from 13 to 17 min and subsequently to 90% of B to 18 min, and held at 90% of B to 20 min. After the analysis, a 10-min post-run program was started at initial conditions in order to equilibrate the analytical column before the next analysis. The autosampler temperature was kept at 4 °C. The injection volume was 3 µL; the flow rate and the column temperature were set at 0.4 mL/min and 50 °C, respectively.

Analyses were performed using the dynamic multiple reaction monitoring (dMRM) acquisition mode. The MS/MS spectra parameters were optimized using source optimizer software Mass Hunter Source Optimizer v. B. 10.1.67 (Agilent Technologies Inc., Santa Clara, CA, USA) while dMRM ion transitions were selected for each compound using Mass Hunter Optimizer software, v. B.07.01 (Agilent Technologies Inc., Santa Clara, CA, USA). After automatic optimization using the abovementioned software, the quality of the obtained results was evaluated manually by additional separate reanalysis of each standard with chosen parameters. The capillary voltage, drying gas flow rate, drying gas temperature, and nebulizer pressure were set at 3500 V, 11 L/min, 340 °C, and 50 psi, respectively.

### Method validation

The method was validated according to the FDA and EMA guidelines in terms of selectivity, linearity, accuracy, precision, matrix effect, recovery, and stability [41, 42].

#### Selectivity

Selectivity was determined by a comparison of blank urine samples with blank urine samples spiked with target analytes at a low-quality-control (LQC) concentration level.

#### Linearity

The linearity of the method was determined based on eight concentration levels, prepared by spiking blank urine samples with appropriate volumes of the working solutions. Then, the sample preparation procedure described in “Preparation of urine and blank urine samples” was applied. The assessed linear ranges of the analytes are shown in Table 2. Calibration curves were performed by plotting the concentrations of the analytes against the relative analyte response (obtained peak area normalized by the peak area of the IS) with the use of MassHunter Workstation software (Agilent Technologies Inc., Santa Clara, CA, USA). Calibration curves were weighted by the factor 1/*y* or 1/*x*.

Sensitivity was assessed based on the designated limit of detection (LOD) and limit of quantification (LOQ), calculated using calibration curve parameters. For each analyte, linear regression was performed with the use of Prism software (GraphPad Software, San Diego, CA, USA).

#### Accuracy and precision

In order to determine the within-run accuracy and precision, quality control samples were prepared at four concentration levels—lower limit of quantification (LLOQ), LQC, medium quality control (MQC), and high quality control (HQC), to cover the calibration curve range. Blank urine was employed for quality control samples in the same manner as patients’ urine samples. Appropriate amounts of the working solutions and blank urine samples were mixed to finally obtain the volume of 100 µL. Then 10 µL of the IS and 900 µL of methanol:water solution (1:1, *v/v*) were added and the samples were prepared as described in “Preparation of urine and blank urine samples.” Six replicates per concentration level were prepared and analyzed. In order to determine the between-run accuracy and precision, sets of QC samples were analyzed in four separate analytical runs, and on separate days. The calculated concentration for accuracy should be within 15% of the nominal concentration, except for LLOQ, for which it can be 20%. The calculated coefficient of variation (CV) for precision should not exceed 15% for QC samples, except for LLOQ, which should not exceed 20%.

#### Matrix effect

The matrix effect should be investigated when mass spectrometric methods are utilized. In the presented study, two sets of samples were prepared. In each set, the matrix effect was assessed at two concentration levels: HQC and LQC. Six replicates were prepared for each level. In the first set, 100 µL of blank urine was mixed with 900 µL of methanol:water solution (1:1, *v/v*) and the samples were evaporated to dryness. After the evaporation, proper amounts of the working solution, IS, and methanol:water solution (1:1, *v/v*) mixture up to 100 µL were added to the dry residue. In the second set, sample preparation was limited to mixing the proper amount of the IS, methanol:water solution (1:1, *v/v*), and the analyte working solution to the final volume of 100 µL. Subsequently, the samples were vortex-mixed and analyzed.

The matrix effect (ME) was assessed as described in the EMA guidelines [41]. Firstly, for each analyte, the matrix factor (MF) was calculated by dividing the peak area in the presence of the matrix by the peak area in the absence of the matrix. The MF was also calculated for the internal standard. Next, the obtained matrix factors were normalized by the MF of the internal standard, and from the obtained IS-normalized matrix factors, the coefficient of variation was determined. The calculated CV should not be greater than 15%.

$$ME=\frac{IS-normalized\;MF\;standard\;deviation}{IS-normalized\;MF\;average}\times100\%$$

#### Stability

Stability was assessed in terms of bench-top, autosampler, and freeze–thaw analyte stability. In stability tests, the prepared samples were analyzed against the freshly prepared calibration curve. To assess stability, the calculated mean concentration was compared to the nominal concentration.

According to the bench-top stability test, two sets of samples, consisting of the blank urine samples spiked with analytes at LQC and HQC levels, were prepared in triplicates. The first set was left at room temperature for 3 h and the second set for 6 h. After that time, 10 µL of the IS and 900 µl of methanol:water solution (1:1, *v/v*) were added and the samples were prepared as described in Sect. 2.5. For the freeze–thaw stability study, three freeze and thaw cycles were performed at HQC and LQC concentration levels in triplicates. The samples were frozen at -20 °C. In the thaw cycles, all samples were thawed at ambient temperature and the first set of samples were prepared as described in “Preparation of urine and blank urine samples.” The remaining samples were refrozen. Such cycles were repeated after 24 h, 48 h, and 72 h.

In the autosampler stability test, three replicates of blank urine samples spiked with analytes at LQC and HQC concentration levels were prepared as described in “Preparation of urine and blank urine samples” and left in the autosampler set at 4 °C for 50 h. The samples were reinjected every 5 h.

#### Recovery

The recovery was determined by comparing the analyte response in the spiked urine samples at LQC, MQC, and HQC concentration levels in six replicates. The responses from samples prepared according to “Preparation of urine and blank urine samples” were compared with those prepared as follows: 100 µL of blank urine was mixed with 900 µL of methanol:water solution (1:1, *v/v*) and evaporated to dryness. Next, to adequate amounts of working solutions, 10 µL of the IS was added and completed up to the final sample volume of 100 µL with the methanol:water solution (1:1, *v/v*).

### Method application to urine samples

The optimized procedure was utilized for the analysis of 133 urine samples collected from patients diagnosed with bladder cancer at 7 time points: 24 h before the TURBT surgery (*n* = 53) and then 24 h (*n* = 50), 2 weeks (*n* = 10), and 3 (*n* = 8), 6 (*n* = 7), 9 (*n* = 3), and 12 (*n* = 2) months after the operation. Urine samples were analyzed against freshly prepared calibration curves. The obtained data were processed with the use of MassHunter Workstation software (Agilent Technologies Inc., Santa Clara, CA, USA). The calculated analyte concentrations were normalized by the creatinine concentration and expressed as micromoles per millimole of creatinine. The concentration of creatinine was measured in external an accredited diagnostics laboratory (Gdańsk, Poland) with the use of spectrophotometric technique.

### Statistical data analysis

The data were described using the following linear mixed-effect model:

$$\mathrm{logDV}\sim \mathrm{TIME}+\mathrm{MET}+\mathrm{MET}:\mathrm{TIME}+(1+\mathrm{MET}|\mathrm{ID})+(1|\mathrm{SAMPLE})\mathrm{sigma}\sim \mathrm{MET}$$

where logDV is the logarithm of measured concentrations (dependent variable) and the model terms are to the right of the tilde character. The model was prespecified and constructed in relation to the proposed experimental design. Since the concentrations of nucleosides and deoxynucleosides were measured at various time points, the model estimates the metabolite, time, and metabolite:time interactions as fixed effects. Consequently, the first terms are fixed effects, such as TIME (time point), MET (analyte), and MET:TIME (interaction). The model also includes two random effects (subjects and sample). The random effects are terms with a bar symbol, such as ID and SAMPLE. The random effect of SAMPLE was used to account for between-sample variations in the diuresis (common variation for all MET). It allowed to control for the confounding effect of diuresis. The random effect of ID was used to account for between-subject variations in MET assuming a correlation between intercepts and slopes. The sigma (standard deviation for within-subject variability) was modelled assuming the fixed effect of MET. The model fitting was done using the brms package (version 2.18.0 Bürkner, 2022) in the free, open-source program R (R Core Team, 2022). The data and complete R codes used in the study are attached as supplementary material.

### Incurred sample reanalysis

According to the EMA guidelines, 10% of the samples (in case the total number of samples is less than 1000) should have been analyzed again during validation process in order to evaluate more accurately the factors affecting the sample during processing and storage. In described research, 15 out of 133 urine samples from randomly selected patients were re-analyzed. EMA criteria indicate that the calculated difference between analyte concentrations determined during the initial and repeat analysis should not exceed 20% in at least 67% of the repeats.

## Results and discussion

Searching for new diagnostic tools, with the use of the metabolomics approach, is of high importance as it may bring essential scientific clarifications, especially in the era of personalized medicine. The main goal of our study was quantitative determination of eleven nucleosides and deoxynucleosides from urine samples obtained from patients before and up to 12 months after bladder cancer resection. Selection of most of the target compounds in our study was based on our previous metabolomics results. The first prior study, performed by Yumba-Mpanga et al. [35], covered the targeted analysis of 17 different metabolites, including two nucleosides: pseudouridine and uridine, in urine collected from BCa patients and healthy volunteers. Samples were analyzed with the use the HPLC-ESI-QqQ/MS technique. The obtained data underwent statistical analysis, and as a result, ten compounds, including two analyzed nucleosides: uridine and pseudouridine were considered as differentiating between compared groups at statistically significant level and consequently were chosen to be analyzed in the described project. The second project covered the targeted analysis of 13 nucleosides in urine (*n* = 248) collected from patients diagnosed with urogenital cancer (*n* = 158) and healthy volunteers (*n* = 95) [36]. Samples were prepared and analyzed with the liquid chromatography-ultraviolet-visible spectrophotometry (LC-UV-Vis) technique based on a validated method. Data obtained from this analysis were reanalyzed by Daghir-Wojtkowiak et al. with the use of multilevel (hierarchical) modeling within the Bayesian method which is considered as more appropriate for large-sample, unbalanced, sex- and age-unadjusted data [37]. As a result 5′-methylthioadenosine was observed as the most associated nucleoside with the occurrence of urogenital cancer. For this reason, it was chosen for the prospective analysis described in the presented project. The third project covered the development and validation of an analytical method for the determination of modified nucleosides in urine samples from healthy volunteers (*n* = 61) and patients suffering from urinary cancer diseases (*n* = 68) with the use of the HPLC-QqQ-MS technique [8]. As a result of data analysis, modified nucleosides, which represent statistically significant differences between studied groups, were selected (N3-methyluridine, inosine, N6-methyladenosine, N2-methylguanosine, N2,N2-dimethylguanosine). Applied multivariate analysis revealed a strong positive correlation between N2-methylguanosine, N2,N2-dimethylguanosine, and N3-methyluridine as well as no correlation between N6-methyladenosine and inosine. High correlation indicates qualitative significant differences in changes of modified nucleosides between patients and controls. Moreover, obtained data was also analyzed by Daghir-Wojtkowiak et al. [16] with the use of a different statistically based approach, including correlations, associations, and interactions. As a result, two nucleosides, N2,N2-dimethylguanosine and N2-methylguanosine, were considered as mostly associated with the development of urogenital cancer, including bladder cancer. As a result of the presented preliminary study, the following additional nucleosides: N3-methyluridine, inosine, N6-methyladenosine, N2-methylguanosine, and N2,N2-dimethylguanosine were selected for the described project.

### Sample preparation

In metabolomics analyses, an important aspect is the development of high-throughput methods that allow for the fast and straightforward analysis of different compounds. Modified nucleosides and deoxynucleosides are found in low concentrations in urine. For this reason, different sample preparation methods, including extraction techniques, were developed in order to concentrate the urine samples so that nucleosides and deoxynucleosides could be accurately quantified in biological matrices. However, each additional sample preparation step increases the total analysis time and decreases the efficiency of the method. In our previous work, we used a commercially available phenyl-boronate:cation exchange mixed-mode solid-phase extraction (SPE) sorbent [38]. However, our further studies have shown that omitting the SPE extraction step gives comparable results in terms of recovery (results not shown) as well as significantly simplifies and shortens the sample preparation time. Therefore, in the presented study, sample preparation was limited to precipitation in methanol:water (1:1 *v/v*) solution and evaporation steps which significantly shorten the time of sample preparation enabling the concurrent analysis of modified nucleosides and deoxynucleosides.

### HPLC-QqQ/MS analysis

The LC conditions including type of chromatographic column, mobile phases, type of elution, gradient pump program, column temperature, and mobile phase flow rate were developed at the early stage of the research. This process was described in our previous study [38]. Briefly, three chromatographic columns were tested all with the same dimensions (3.0 × 100 mm, particle size 3.5 µm), namely Zorbax Eclipse Plus C18, Zorbax Sb–C3, and Zorbax Sb-Aq. The best separation and reproducible retention of the determined analytes were obtained with the use of Zorbax Sb-Aq (3.0 × 100 mm, 3.5 µm) which is alkyl reversed-phase bonded stationary phase. This column is suitable to retain hydrophilic compounds at even highly aqueous mobile phase composition such as 100% of water. The column was designed using Stable Bond technology which avoids exhibiting the so-called phase collapse that normally can be observed in conventional C18 stationary phases in the highly aqueous mobile phase. In the present study, the gradient starts only from 1% of the aqueous mobile phase (0.1% of formic acid in deionized water) which allowed the most hydrophilic nucleosides like Pse and U to be retained on the chromatographic column. The retention time of all the determined compounds was reproducible throughout the validation process as well as urine sample determination. In regard to mass spectra conditions, the ESI source parameters, capillary, fragmentor and collision cell energy voltages were optimized. The capillary voltage, drying gas flow rate, drying gas temperature, and nebulizer pressure were set at 3500 V, 11 L/min, 340 °C, and 50 psi, respectively. The obtained dynamic MRM transitions (quantifier and qualifier ion transitions, collision energy, selected fragmentor voltages) are presented in Table 1. Analyte structures and their molecular weights are given in Supplementary Materials (Table SM2), while product ion mass spectra along with a proposed fragmentation pattern are provided in Figure SM1. The selected transitions have been also confirmed based on literature data [46–48]. The fragmentation pattern of all analytes except Pse consisted in the decomposition of precursor ion to protonated base (BH^+^) by the loss of the neutral 2′-deoxyribose moiety (116u) in the case of deoxynucleosides or ribose moiety (132u) in the case of nucleosides. It is the characteristic fragmentation pattern of nucleosides and deoxynucleosides. Pseudouridine, in contrast to other nucleosides, has a C–C bond between the nucleobase and sugar moiety instead of C1′ (ribose)-N1 (uracil) linkage, which is the main cause of fragmentation pattern differences. Therefore, product ions are created through the loss of one, two, or three water molecules as well as various fragments derived from both ribose and base moiety (Figure SM1). Besides, it has to be underlined that in most cases the quantifiers and qualifiers were chosen based on their highest ion abundances (protonated base and further fragmentation of protonated base). However, due to the very efficient ionization and high concentration in urine samples of two nucleosides, Pse and 2,2dmG, their product ions were selected according to the second and third most abundant ions which decreased their abundances in analyzed urine samples.

**Table 1:** LC/MS/MS parameters obtained after collision-induced dissociation in dynamic MRM mode

Compound	*t*_R_ [min]	Precursor ion [*m/z*]	Product ion [*m/z*]		CE [V]		Fragmentor voltage [V]
			Quantifier ion	Qualifier ion	Quantifier ion	Qualifier ion	
Pse	1.9	245	191	167	20	10	90
U	2.6	245	113	70	10	35	70
Ino	4.3	269	137	119	10	40	80
3mU	5.9	259	127	96	5	40	80
2dG	6.7	268	152	135	10	40	80
2mG	8.0	298	166	149	40	20	90
8OH2dG	9.5	284	168	140	10	35	80
6mA	9.9	282	150	123	40	40	110
2,2dmG	11.3	312	110	135	30	40	80
8BrG (IS)	11.7	362	230	213	20	40	100
5-MTA	14.3	298	136	119	20	40	90
MTA	16.7	314	182	134	20	40	80

*CE* collision energy, *t*_*R*_ retention time, *Pse* pseudouridine, *U* uridine, *Ino* inosine, *3mU* N3-methyluridine, *2dG* 2-deoxyguanosine, *2mG* N2-methylguanosine, *8OH2dG* 8-hydroxy-2-deoxyguanosine, *6mA* N6-methyladenosine, *2,2dmG* N2,N2-dimethylguanosine, *8BrG* 8-bromoguanosine (*IS* internal standard), *5-MTA* 5-deoxy-5-methylthioadenosine, *MTA* 2-methylthioadenosine

### Method validation

**Fig. 1:**
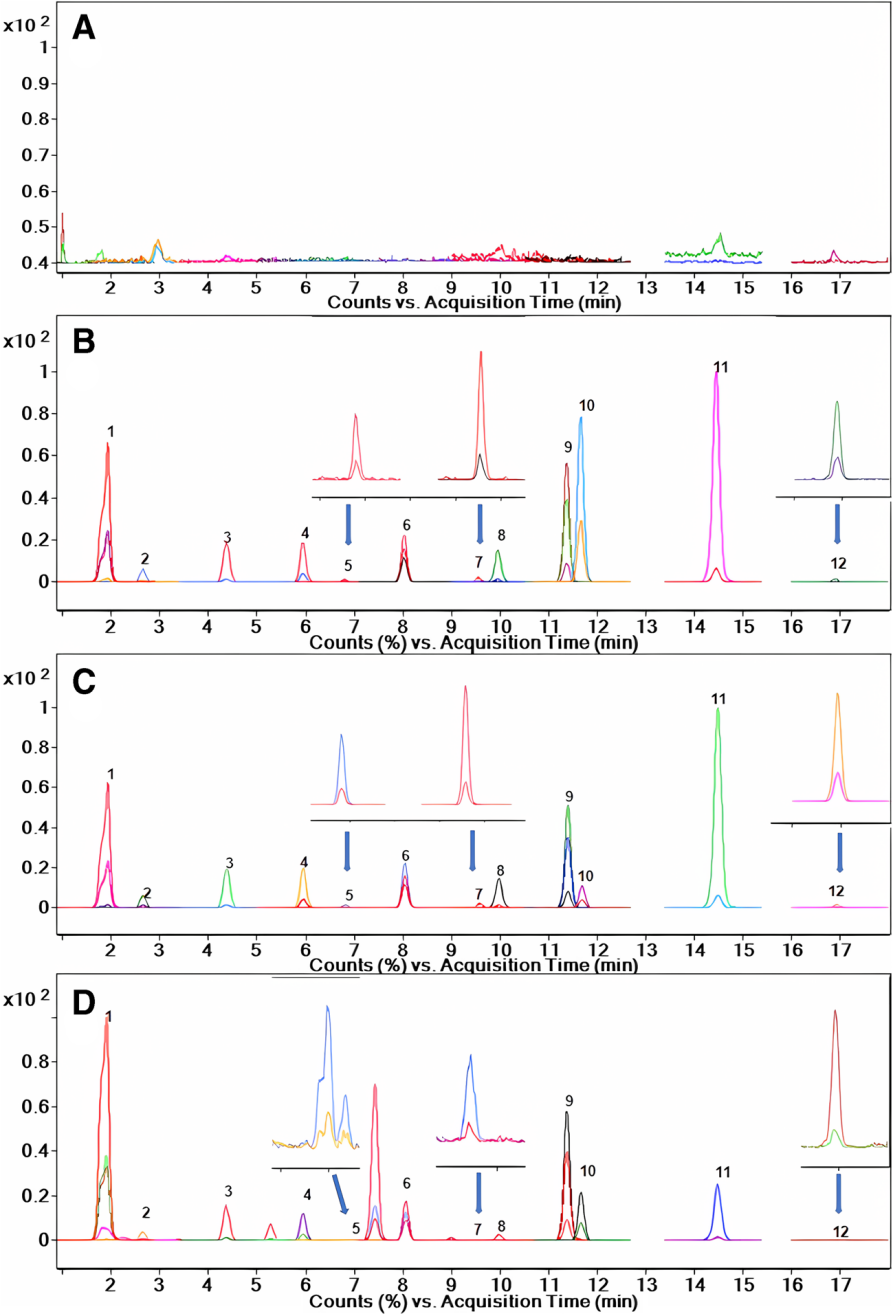
The exemplary chromatograms of **A** blank urine, **B** blank urine spiked with analytes at LQC, and **C** HQC concentration levels as well as **D** an exemplary urine sample derived from a bladder cancer patient. 1) Pse (*t*_R_ = 1.9 min); 2) U (*t*_R_ = 2.6 min); 3) Ino (*t*_R_ = 4.3 min); 4) 3mU (*t*_R_ = 5.9 min); 5) 2dG (*t*_R_ = 6.9 min); 6) 2mG (*t*_R_ = 8.0 min); 7) 8OH2dG (*t*_R_ = 9.5 min); 8) 6mA (*t*_R_ = 9.9 min); 9) 2,2dmG (*t*_R_ = 11.3 min); 10) 8BrG (IS) (*t*_R_ = 11.7 min); 11) 5-MTA (*t*_R_ = 14.3 min); 12) MTA (*t*_R_ = 16.7 min). The *Y* axis is expressed as each peak relative abundance

Quantitative determination of endogenous compounds is a great challenge. Therefore, it was decided to use synthetic urine matrix (Surine® negative urine control) that is free of nucleosides but has the chemical composition and physicochemical properties common to human urine samples. The synthetic urine samples were fortified using an adequate amount of nucleoside standards to create the calibration curve according to which the concentration of analytes in urine samples was calculated. Additionally, in terms of selectivity, the method was validated and proved artificial urine matrix to be free of nucleoside targets (Fig. 1A). Besides, to minimize the possible analytical variability that may occur at the sample preparation and analytical determination steps, the internal standard (8BrG) was added to all samples during the sample preparation procedure. The choice of 8BrG was based on our previous experience with this compound [8, 36, 49, 50]. We are aware that the usage of deuterated standards for each analyte is currently the strategy of choice when using mass spectrometry detection. Concerning nucleosides and deoxynucleosides, only some of them have stable isotopes commercially available. Therefore, we decided to use non-deuterated IS (8BrG) which has a common main structure with nucleosides but has an exogenous origin. Besides, according to our previous studies, nucleosides, including 8-BrG, are stable when fortified into urine samples [50].

#### Selectivity

The evaluation of selectivity was performed by a comparison of blank urine samples with blank urine samples spiked with analytes at LQC concentration level. In Fig. 1, exemplary chromatograms of blank urine samples (Fig. 1A) as well as blank urine samples spiked with analytes at LQC concentration level (Fig. 1B) are presented. Additionally, in Fig. 1C, D, blank urine samples spiked with analytes at HQC concentration level as well as urine samples derived from patients are shown.

#### Calibration curves

Calibration curves for each analyte were obtained by plotting the analyte’s nominal concentration with the analyte’s relative response. Concentration ranges were chosen during pre-validation analyses and based on expected concentrations in urine samples. The results are presented in Table 2. The linearity was validated in a specific range taking into account the possible concentration of each nucleoside in urine samples. The linearity was set as follows: 2.5–250 µM for Pse; 0.25–2.5 µM for 2,2dmG; 0.1–10 µM for 2mG; 0.05–5 µM for U, I, and 3mU; 0.025–2.5 µM for 5-MTA; 5–500 nM for 6mA; 1–100 nM for 2dG; and 0.25–25 nM for MTA. The coefficient of variation of linear calibration curves was in a range from 0.987 to 0.998. Based on the calculated slope and the standard deviation of the intercept, LOD and LOQ were assessed. For nearly all nucleosides, LOQ was lower than nominal LLOQ, except for 2-deoxuguanosine, 8-hydroxy-2-deoxyguanosine, and 2-methylthioadenosine, where LOQ was at the LLOQ level. As it is presented in Table 2, the LOQ was calculated to be in a range from 0.25 nM for MTA to 1.546 µM for Pse. The optimized and validated LC-QqQ/MS quantitative method was assessed to be sensitive enough to cover all variations in analyte concentrations that were measured in studied urine samples derived from bladder cancer patients.

**Table 2:** Linear parameters: linear range, weight error, slope, standard deviation, and confidence interval calculated for slope as well as for intercept, correlation coefficient, residual standard deviation, limit of detection, and limit of quantification for analyzed nucleosides and deoxynucleosides

Analyte	Linear range [µM]	Weight error	Slope	SD for slope	Confidence interval (95%) for slope	Intercept	SD for intercept	Confidence interval (95%) for intercept	Correlation coefficient	S*xy*	LOD [µM]	LOQ [µM]	Ion ratio (uncertainty range)
Pse	2.5–250	1/*y*	0.08	0.001	0.074–0.079	0.008	0.012	− 0.016 to 0.033	0.996	0.043	0.510	1.546	17.1 (14.0–20.9)
U	0.05–5	1/*y*	0.29	0.004	0.280–0.295	0.0012	0.0009	− 0.0006 to 0.003	0.996	0.003	0.001	0.030	12.4 (9.0–13.6)
I	0.05–5	1/*y*	1.03	0.017	0.995–1.068	0.011	0.004	0.002 to 0.020	0.995	0.013	0.013	0.040	9.3 (7.6–11.4)
3mU	0.05–5	1/*y*	0.97	0.009	0.951–0.990	0.004	0.002	− 0.0007 to 0.008	0.998	0.008	0.007	0.022	17.8 (14.2–21.3)
2dG	0.001–0.1	1/*x*	2.89	0.066	2.745–3.032	0.001	0.0003	2.745 to 3.032	0.993	0.0008	0.0004	0.001	35.8 (29.7–44.6)
2mG	0.1–10	1/*y*	0.54	0.008	0.521–0.555	0.011	0.004	0.003 to 0.019	0.996	0.012	0.023	0.070	86.5 (67.9–101.8)
8OH2dG	0.0025–0.25	1/*x*	2.14	0.046	2.043–2.232	− 0.0005	0.0005	− 0.002 to 0.0006	0.990	0.002	0.0008	0.0025	31.1 (23.2–34.8)
6mA	0.005–0.5	1/*y*	7.12	0.131	6.849–7.399	0.006	0.003	5.106e − 006 to 0.013	0.993	0.010	0.001	0.004	14.0 (11.1–16.7)
2,2dmG	0.25–25	1/*y*	0.48	0.006	0.470–0.498	0.055	0.008	0.037 to 0.072	0.998	0.021	0.053	0.162	8.8 (6.9–10.3)
5-MTA	0.025–2.5	1/*y*	11.80	0.171	11.440–12.150	0.047	0.020	0.005 to 0.089	0.996	0.066	0.006	0.017	8.1 (6.3–9.5)
MTA	0.00025–0.025	1/*x*	15.22	0.368	14.450–15.980	0.0006	0.0004	− 0.0003 to 0.002	0.987	0.001	0.00009	0.00025	51.2 (39.0–58.4)

*SD* standard deviation, *Sxy* residual standard deviation, *LOD* limit of detection, *LOQ* limit of quantification, *Pse* pseudouridine, *U* uridine, *Ino* inosine, *3mU* N3-methyluridine, *2dG* 2-deoxyguanosine, *2mG* N2-methylguanosine, *8OH2dG* 8-hydroxy-2-deoxyguanosine, *6mA* N6-methyladenosine, *2,2dmG* N2,N2-dimethylguanosine, *8BrG* 8-bromoguanosine (*IS* internal standard), *5-MTA* 5-deoxy-5-methylthioadenosine, *MTA* 2-methylthioadenosine

#### Accuracy and precision

Results obtained during the validation process are presented in Table 3. Accuracy was given in percent and expressed as a percentage of the obtained concentration relative to the nominal concentration. Intra-day accuracy was in the range of 92.5–108.4%, while inter-day accuracy was in the range of 93.1–109.4%. The obtained results met the EMA and FDA criteria. In the case of within-run, as well as between-run, accuracy, the mean concentration for LLOQ was within 15 or 20% of the nominal concentration.

**Table 3:** Results of method validation: intra- and inter-day precision, accuracy, recovery, and matrix effect

Compounds	QC samples	Nominal concentration [µM]	Intra-day (*n* = 6)		Inter-day (*n* = 6)		Recovery (*n* = 6) [%]	Matrix effect (*n* = 6) CV [%]
			Accuracy [%]	Precision CV [%]	Accuracy [%]	Precision CV [%]		
2,2dmG	LLOQ	0.25	95.99	9.00	95.26	8.98		
	LQC	1.25	107.40	1.02	109.39	2.88	89.38	7.98
	MQC	5	104.59	3.21	106.52	3.98	96.01	
	HQC	10	98.90	2.89	103.59	5.37	87.98	12.24
2dG	LLOQ	0.001	98.25	8.69	98.29	6.83		
	LQC	0.005	99.61	6.89	102.64	5.56	89.87	9.55
	MQC	0.02	96.47	4.57	99.67	5.14	95.75	
	HQC	0.04	92.45	2.83	98.89	5.96	87.32	13.73
5-MTA	LLOQ	0.025	95.36	6.39	95.91	8.17		
	LQC	0.125	97.18	2.70	105.50	5.71	89.16	8.47
	MQC	0.5	105.93	3.88	107.89	3.90	95.46	
	HQC	1	99.74	3.53	103.13	3.41	86.67	13.52
8OH2dG	LLOQ	0.0025	101.83	7.12	101.88	10.49		
	LQC	0.0125	94.06	4.21	93.08	8.88	68.50	9.76
	MQC	0.05	96.25	6.07	99.94	6.75	79.08	
	HQC	0.1	93.64	4.48	97.43	8.21	76.35	11.17
Ino	LLOQ	0.05	97.96	6.07	98.75	6.68		
	LQC	0.25	101.59	1.89	103.87	3.39	87.52	9.99
	MQC	1	98.48	3.56	102.52	4.77	97.35	
	HQC	2	95.52	2.63	101.86	6.43	84.52	14.28
6 mA	LLOQ	0.005	95.20	8.36	99.08	8.86		
	LQC	0.025	103.26	3.83	106.83	5.73	90.82	8.48
	MQC	0.1	108.41	2.23	109.31	2.81	95.42	
	HQC	0.2	103.22	2.58	105.17	2.47	87.86	11.36
2mG	LLOQ	0.1	95.04	9.26	95.69	7.99		
	LQC	0.5	102.62	1.34	107.34	3.87	89.04	7.96
	MQC	2	100.43	2.80	105.94	4.76	96.48	
	HQC	4	97.22	3.04	103.47	5.59	88.45	11.17
MTA	LLOQ	0.00025	99.97	8.09	98.95	8.51		
	LQC	0.00125	104.48	2.89	104.01	4.48	92.24	5.72
	MQC	0.005	103.18	4.53	102.33	5.00	95.95	
	HQC	0.01	101.23	2.56	102.01	2.63	87.13	11.47
3-MU	LLOQ	0.05	99.30	6.78	100.56	6.75		
	LQC	0.25	101.54	1.42	102.31	3.24	87.86	10.45
	MQC	1	98.21	3.75	100.80	4.48	96.67	
	HQC	2	95.54	3.33	100.86	6.42	84.92	14.96
Pse	LLOQ	2.5	96.20	9.01	96.36	7.76		
	LQC	12.5	106.52	0.59	108.51	5.19	87.15	9.74
	MQC	50	102.54	2.88	106.43	4.23	95.97	
	HQC	100	96.88	3.34	105.73	9.81	85.67	12.48
U	LLOQ	0.05	99.32	7.87	99.23	8.08		
	LQC	0.25	103.78	2.09	103.45	5.22	85.68	10.04
	MQC	1	99.23	2.97	101.81	4.32	97.32	
	HQC	2	96.17	3.34	102.68	6.97	82.72	14.21

*LLOQ* lower limit of quantification, *LQC* low concentration of quality control samples, *MQC* medium concentration of quality control samples, *HQC* high concentration of quality control samples, *2,2dmG* N2,N2-dimethylguanosine, *2dG* 2-deoxyguanosine, *5-MTA* 5-deoxy-5-methylthioadenosine, *8OH2dG* 8-hydroxy-2-deoxyguanosine, *Ino* inosine, *6 mA* N6-methyladenosine, *2mG* N2-methylgoanosine, *MTA* 2-methylthioadenosine, *3 mU* N3-methyluridine, *Pse* pseudouridine, *U* uridine

Precision was expressed as CV [%]. According to the EMA and FDA guidelines, calculated inter- as well as intra-day precision should not exceed 15%, except for LLOQ, where it should not exceed 20%. As shown in Table 3, the obtained data met these criteria. Within-run precision was in the range of 0.6–9.3%, and between-run precision was in the range of 2.5–10.5%.

#### Matrix effect

The matrix effect was assessed according to the EMA guidelines as described in Sect. 1.1.5 to evaluate the influence of urine components on ion suppression. It was expressed as the CV of the IS-normalized matrix factor. The calculated CV should not exceed 15%. The results are presented in Table 3. The calculated CV for all the analyzed nucleosides were within the acceptable range of 7.96–14.96%.

#### Stability

Stability was tested in the case of bench-top stability, freeze-thaw stability, and autosampler stability. Results from the stability evaluation are presented in Table 4. All stability results were expressed as a percentage of the obtained concentration relative to the nominal concentration. The EMA guidelines recommend that the calculated concentration should be within ± 15% of the nominal concentration. Time points chosen for bench-top stability (3 and 6 h) covered the maximum duration of sample preparation. Although the sample preparation procedure does not include a time-consuming extraction method, it consists of an evaporation step which lasts around 1.5 h. As it can be seen in Table 4, the results obtained for all analytes met the requirements at both concentration levels, after 3 as well as 6 h. After 3 h, the calculated percentage was in the range of 95.0–104.3%, while after 6 h, it was in the range of 86.2–112.3%.

**Table 4:** Results of validation regarding compound stability in various conditions

Compound	QC samples	Nominal concentration [µM]	Bench top stability (*n* = 3)		Autosampler stability (*n* = 3)			Freeze/thaw stability (*n* = 3)			
			3 h [%]	6 h [%]	0 h [%]	25 h [%]	50 h [%]	0 cycle [%]	1st cycle [%]	2nd cycle [%]	3rd cycle [%]
2,2dmG	LQC	1.25	111.04	112.32	106.59	104.85	104.60	115.12	121.86	116.41	114.51
	HQC	10	104.25	93.21	102.67	102.52	103.22	106.13	112.25	110.97	112.81
2dG	LQC	0.005	103.90	101.02	91.55	94.37	99.02	110.78	110.58	111.47	111.24
	HQC	0.04	96.02	86.21	92.98	92.23	90.21	107.97	113.71	107.61	107.49
5-MTA	LQC	0.125	104.66	104.24	96.08	91.90	91.48	107.93	111.57	107.10	106.40
	HQC	1	101.25	91.49	95.99	93.62	93.89	99.63	107.99	104.46	105.93
8OH2dG	LQC	0.0125	113.00	108.07	112.74	106.89	107.72	97.03	105.46	110.94	114.06
	HQC	0.1	101.58	92.51	103.13	106.62	103.65	93.20	89.57	100.32	106.97
Ino	LQC	0.25	107.85	108.22	107.18	105.20	109.96	114.87	115.14	112.41	113.30
	HQC	2	101.67	91.20	107.80	108.37	108.55	111.93	112.07	107.81	112.32
6mA	LQC	0.025	107.54	106.93	97.70	92.10	88.91	106.34	114.34	115.82	113.13
	HQC	0.2	103.56	94.41	93.67	89.46	88.22	98.13	114.44	108.34	104.99
2mG	LQC	0.5	112.89	108.82	108.20	106.51	104.44	114.75	115.87	116.39	111.50
	HQC	4	102.58	91.13	101.06	100.60	100.43	108.75	113.85	110.30	109.14
MTA	LQC	0.00125	102.35	98.17	101.00	99.67	103.43	92.85	111.82	114.48	109.86
	HQC	0.01	104.0	92.01	90.03	97.67	104.10	91.76	112.14	112.88	98.68
3mU	LQC	0.25	106.20	100.70	105.41	106.82	108.29	114.33	112.91	114.86	113.48
	HQC	2	95.56	90.04	105.51	99.78	100.32	108.13	112.91	103.97	109.38
Pse	LQC	12.5	107.69	105.75	105.39	107.19	107.60	108.24	115.87	115.83	114.11
	HQC	100	95.01	86.41	90.13	95.42	95.52	96.86	103.64	102.35	106.32
U	LQC	0.25	108.85	108.05	107.51	107.78	107.39	111.92	114.42	115.20	112.28
	HQC	2	101.46	92.43	99.02	101.75	104.23	106.39	113.70	108.05	111.31

*LQC* low concentration of quality control samples, *HQC* high concentration of quality control samples, *2,2dmG* N2,N2-dimethylguanosine, *2dG* 2-deoxyguanosine, *5-MTA* 5-deoxy-5-methylthioadenosine, *8OH2dG* 8-hydroxy-2-deoxyguanosine, *Ino* inosine, *6mA* N6-methyladenosine, *2mG* N2-methylguanosine, *MTA* 2-methylthioadenosine, *3mU* N3-methyluridine, *Pse* pseudouridine, *U* uridine

Freeze-thaw stability included the evaluation of analyte stability in a urine matrix kept at  − 20 °C and then brought to ambient temperature. Three cycles of freeze-thaw were performed. Cycle 0 reference samples were prepared freshly. As shown in Table 4, almost all analytes met the EMA requirements. For some compounds (N2,N2-dimethylguanosine, inosine, N6-methyladenosine, N2-methylguanosine, pseudouridine, and uridine), the results were above 15%. Such deviations were observed after the 1^st^ and 2^nd^ thawing-freezing cycles, never after the 3^rd^ cycle, and always at the level of LQC concentrations. Moreover, for three compounds, these deviations were observed after more than one cycle. For N2,N2-dimethylguanosine, results above 15% were obtained after the 0, 1^st^, and 2^nd^ cycles, and for N2-methylguanosine and pseudouridine after the 1^st^ and 2^nd^ cycles. The obtained results may indicate that guanosine nucleosides, especially at the low concentration level, are sensitive to the thawing and freezing processes, which should be avoided when working with such compounds. It should be emphasized that the urine samples analyzed in this study were frozen at  − 80 °C after collection from patients and then thawed once, during the method application step.

Samples under autosampler stability tests were kept in the autosampler at 4 °C for 50 h and analyzed at 0-h, 25-h, and 50-h time points. In Table 4, the results from stability studies are presented. Such a long time of stability evaluation was due to the single sample analysis duration (20 min plus 10 min post time) as well as the high number of urine samples that were planned to be analyzed (*n* = 133). In order not to split the urine sample chromatographic analysis step into many batches, 50-h autosampler stability was tested. As presented in Table 4, all analytes passed the EMA requirements.

#### Recovery

Recovery was examined to evaluate the efficiency of the sample preparation procedure. For all the compounds but 8-hydroxy-2-deoxyguanosine, the recovery was in the range of 82.7 to 97.3%. However, for 8-hydroxy-2-deoxyguanosine, the resulting recovery was in the range of 68.5–79.1%. Although the recovery of 8-hydroxy-2-deoxyguanosne was lower than those of other metabolites, the detected concentration of this compound in real urine samples was above LOQ. Additionally, we calculated the repeatability of the recovery studies, which is also important in analysis quality assessment. The repeatability for 8-hydroxy-2-deoxyguanosne was in the range of 3.13–10.92%, which passes the FDA criteria.

#### Sample purity

The assessment of compound purity is crucial when clinical and pre-clinical samples are analyzed. According to the European Commission decision, it is recommended to measure ion ratios to ensure peak purity [51]. In the present study, the obtained data were evaluated in terms of ion ratio parameter using Quantitative MassHunter Workstation software. Using this software, the calculation of the ratios of the quantitative ions’ peak area to the peak area of the qualitative ions was carried out. As a result, the peak area of each qualitative ion constituted 80% of the peak area of quantitative ion with 20% of uncertainty range. The calculations were made among all determined samples and met the required criteria listed in the Commission’s decision, which proves the purity of the analyzed peaks. The ion ratios followed in the analysis are presented in Table 1.

### Application to urine samples

The validated method was used to analyze urine samples from patients diagnosed with bladder cancer undergoing TURBT surgery. Fifty-three patients were enrolled in the study. Urine samples were collected at 7 time points: 24 h before TURBT (*n* = 53), 24 h after TURBT (*n* = 50), and then 2 weeks (*n* = 10) and 3 (*n* = 8), 6 (*n* = 7), 9 (*n* = 3), and 12 (*n* = 2) months after surgery. The aim was to verify whether changes in the concentration of the analyzed nucleosides and deoxynucleosides were associated with the presence of the bladder tumor and the time after tumor resection. Samples were prepared as described in “Preparation of urine and blank urine samples” and were analyzed against freshly prepared calibration curves. Then, the designated concentrations were normalized to the creatinine concentration. The obtained results are presented in Table 5.

**Table 5:** Nucleoside/creatinine ratios [µM nucleoside/mM creatinine] in the urine samples measured at different time points before and after TURT surgery

Compound	Before TURT (*n* = 53)	24 h after TURT (*n* = 50)	2 weeks after TURT (*n* = 10)	3 months after TURT (*n* = 8)	6 months after TURT (*n* = 7)	9 months after TURT (*n* = 3)	12 months after TURT (*n* = 2)	
	Mean ± SD	Mean ± SD	Mean ± SD	Mean ± SD	Mean ± SD	Mean ± SD	Min	Max
Pse	36.76 ± 24.90	31.09 ± 22.63	42.35 ± 30.23	41.42 ± 27.47	43.02 ± 16.37	47.17 ± 39.66	19.87	27.43
U	0.20 ± 0.14	0.22 ± 0.18	0.21 ± 0.16	0.21 ± 0.14	0.27 ± 0.14	0.16 ± 0.08	0.08	0.11
I	0.16 ± 0.12	0.23 ± 0.22	0.21 ± 0.20	0.14 ± 0.14	0.16 ± 0.10	0.24 ± 0.24	0.12	0.15
3mU	0.21 ± 0.14	0.15 ± 0.11	0.21 ± 0.11	0.24 ± 0.12	0.23 ± 0.10	0.22 ± 0.12	0.12	0.15
2dG*	1.70 ± 1.38	1.09 ± 1.08	2.00 ± 1.78	1.25 ± 0.77	1.40 ± 0.91	1.39 ± 0.85	0.51	1.03
2mG	0.78 ± 0.54	0.51 ± 0.34	0.71 ± 0.44	1.00 ± 0.86	0.93 ± 0.55	1.01 ± 0.49	0.44	0.50
8OH2dG*	2.53 ± 1.39	1.65 ± 1.22	2.94 ± 1.64	3.34 ± 2.22	3.56 ± 1.44	3.10 ± 2.55	1.53	1.78
6mA	0.012 ± 0.011	0.028 ± 0.073	0.024 ± 0.036	0.027 ± 0.046	0.011 ± 0.007	0.008 ± 0.004	0.005	0.007
2,2dmG	2.11 ± 1.37	1.68 ± 0.93	2.00 ± 0.86	2.30 ± 1.03	2.59 ± 0.82	2.12 ± 1.05	1.44	1.48
5-MTA*	0.08 ± 0.06	0.08 ± 0.12	0.12 ± 0.14	0.11 ± 0.09	0.06 ± 0.03	0.07 ± 0.06	0.03	0.06
MTA	0.47 ± 0.42	0.20 ± 0.20	0.61 ± 0.55	0.72 ± 0.66	0.56 ± 0.50	0.92 ± 0.91	0.55	0.90

*SD* standard deviation, *Pse* pseudouridine, *U* uridine, *Ino* inosine, *3mU* N3-methyluridine, *2dG* 2-deoxyguanosine, *2mG* N2-methylguanosine, *8OH2dG* 8-hydroxy-2-deoxyguanosine, *6mA* N6-methyladenosine, *2,2dmG* N2,N2-dimethylguanosine, *8BrG* 8-bromoguanosine (*IS* internal standard), *5-MTA* 5-deoxy-5-methylthioadenosine, *MTA* 2-methylthioadenosine

^*^Concentrations are expressed as µM nucleoside/mmol creatinine except for 2dG, 8OH2dG, and MTA where concentrations are expressed as nM nucleoside/nnmol creatinine. Due to the small number of samples, for the last time point, the minimum and maximum values are given instead of the average concentration

### Data analysis

**Fig. 2:**
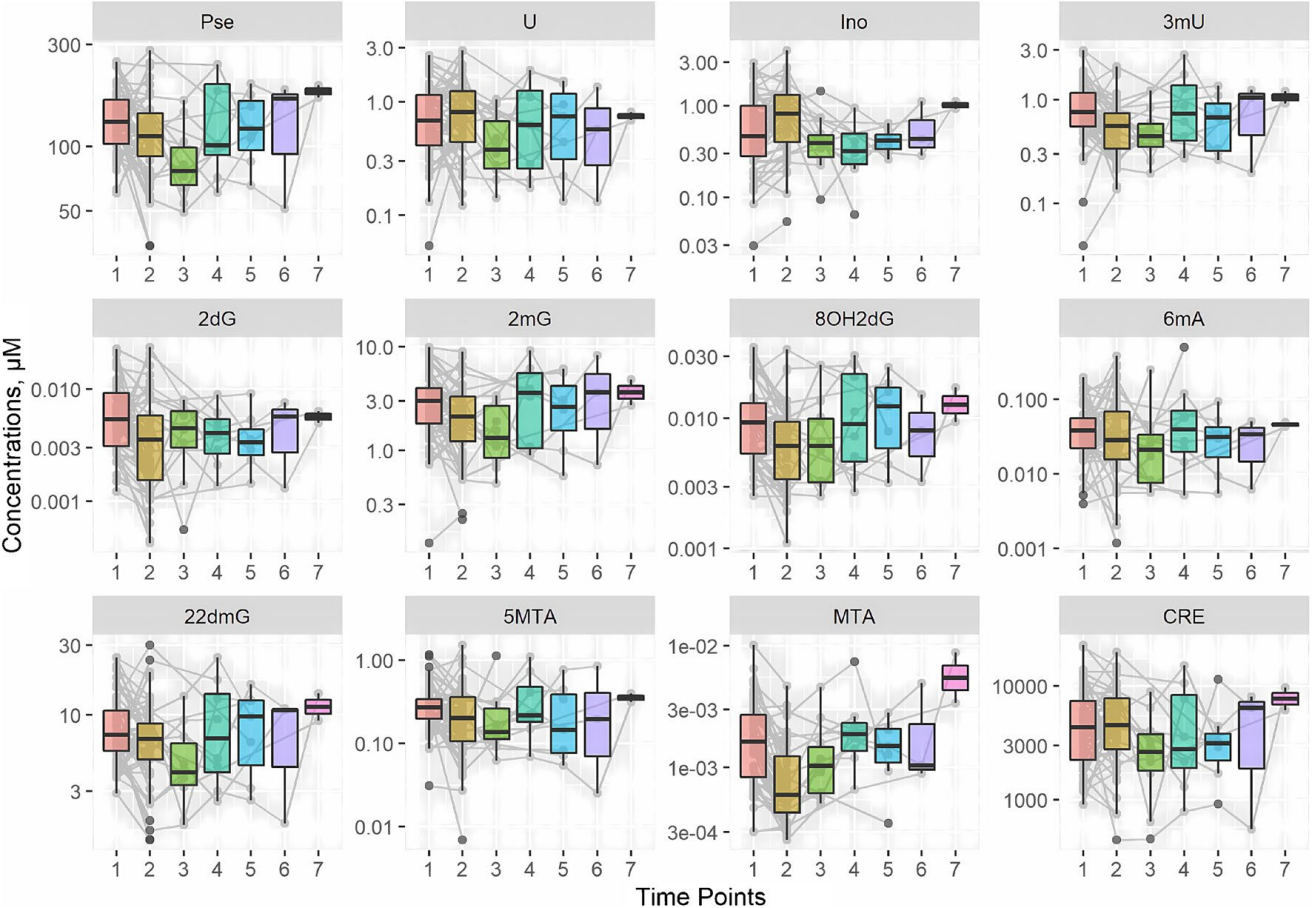
Raw data stratified by MET. Individual measurements are shown in gray. The boxplot summarizes all the measurements collected at a particular time point

**Fig. 3:**
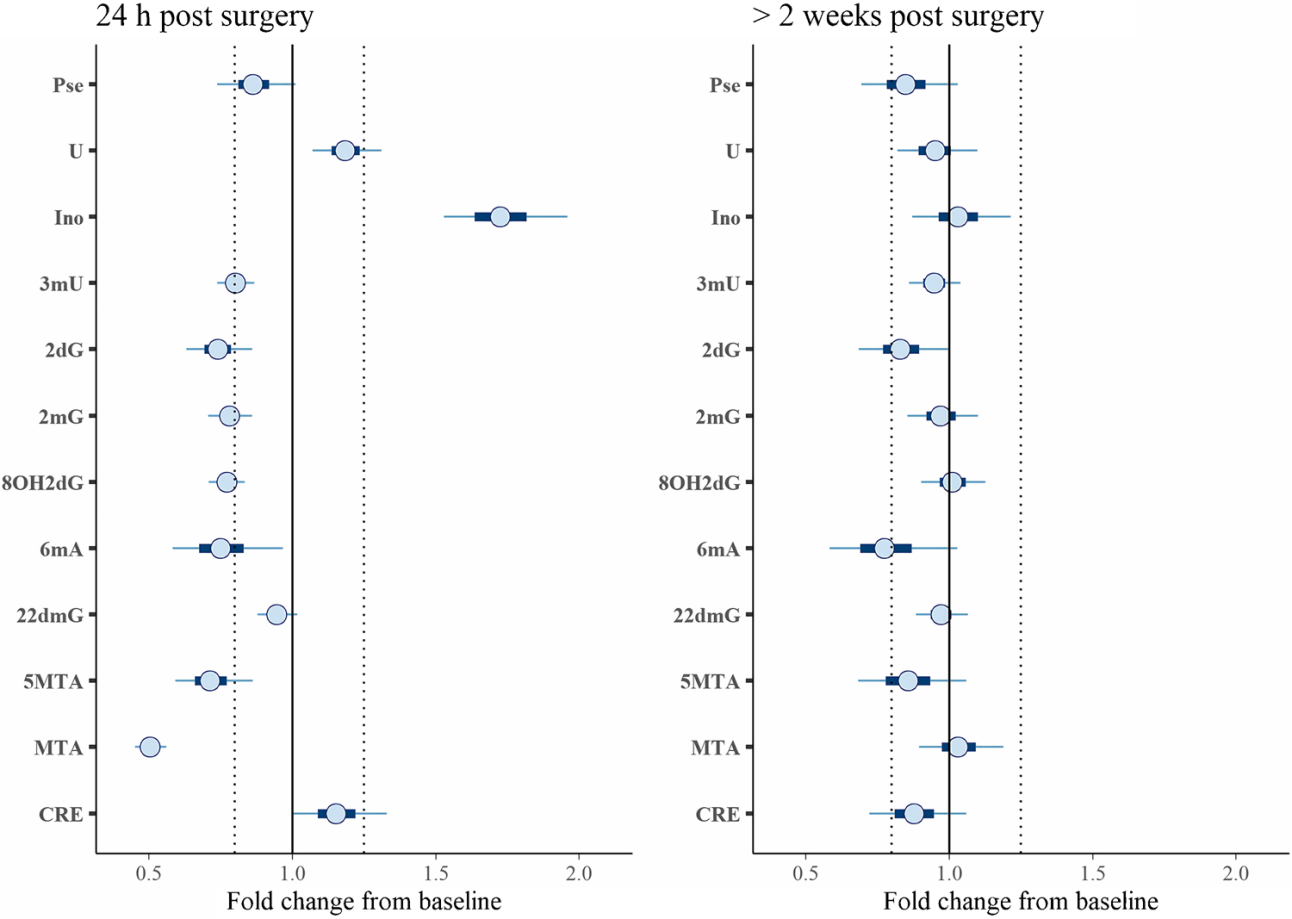
Forest plot of the estimated difference in MET concentrations for a typical subject and typical diuresis. Horizontal bars/lines represent 50%/95% credible intervals. The vertical dashed lines indicate 0.8- and 1.25-fold differences (approximately corresponding to 20% change)

The data set was analyzed using a linear mixed-effect model that took into account the nested structure of the measurements and various levels of variations. The main limitation of the study is the collection of only a few urine samples at the last time points, e.g., only 2 and 3 samples were collected 12 and 9 months after tumor resection. This was handled by assuming constant average concentrations of metabolites for time points 3–7 for each individual. Figure 2 presents the raw data. Figure 3 presents the estimated change from the baseline in the concentration of each MET.

As it can be noticed from Fig. 3, the greatest difference in the concentration of nucleosides was observed between the 1^st^ and 2^nd^ points, thus before and 24 h after tumor resection. A decreased level in the 2^nd^ time point was observed for Pse, 3mU, 2dG, 2mG, 8OH2dG, 6mA, 22mG, 5MTA, and MTA, whereas in the case of Ino and U, the level was increased. The metabolites 2,2mG and 2mG, 5-MTA, and 6mA were previously statistically calculated and proposed as tentative urogenital tract cancer markers in our other studies [8, 16, 37]. For MTA and Ino, a large negative (0.5-fold lower concentration) and positive (1.7-fold higher concentration) effect were observed 24 h post-surgery, respectively.

The decreased levels of nucleosides after tumor resection seems to be in agreement with the thesis that elevated levels of urinary nucleosides can be related with tumor occurrence and can be decreased after its resection [3]. However, this thesis would also require the concentrations of nucleosides to be decreased at further time points. Here, the determined concentrations increased or varied along with the subsequent time points. Moreover, it should be underlined that the lack of a control group also limits the possibility to answer whether there is an effect of cancer removal on the urine concentration of nucleosides and deoxynucleosides. It is possible that the observed differences are related to the surgical procedure itself. Therefore, a longer follow-up study should be continued covering those patients who underwent tumor resection but were not included in the latter time points of the project. In regard to the elevated inosine level which was observed after tumor resection, this might be associated with dysregulated purine metabolism in the cancer state and inosine release from tumor cells. The study of Chen et al. revealed that inosine can be released from dead or dying cells [52, 53]. Then, this metabolite likely induces adenosine receptors that promote proliferation of other survived tumor cells. Changes in inosine levels might be associated with dysregulated purine metabolism in the cancer state. However, it should be also stressed that surgical operation itself could induce such changes in the urine levels of target metabolites, like mechanistic damage of cancer cells. Still, confirmatory studies in the applied model with healthy patients are unattainable.

The decreased level of the second metabolite (2-methylthioadenosine, MTA) can also be related with some changes in purine metabolism, but the mechanism of its role as potential cancer indicator remains unclear.

The concept of a longitudinal monitoring of selected potential targets of bladder cancer was recently published by Carvalho et al. [54]. The study used proteomic tools in the search for beneficial bladder cancer biomarkers allowing for optimization of invasive procedures such as cystoscopy. Researchers monitored patients included in the study for a period of over 5 years, proving that their approach was beneficial in comparison to shorter studies. The presented analytical method, due to its simplicity, could be an effective approach in longitudinal studies taking into account monitoring of selected nucleosides and deoxynucleosides as promising indicators of the BCa disease.

### Incurred sample reanalysis (ISR)

According to the EMA guidelines, ISR experiment is recommended especially in pre-clinical studies and in the early stages of clinical trials. This guideline suggests that 10% of the samples should be reanalyzed if the studied samples are less than 1000. This parameter was also previously assessed in targeted metabolomics studies [55, 56]. However, it is not commonly determined during the quantitative method validation for endogenous compounds. To the authors’ best knowledge, the parameter was not determined in the published methods for the quantitative determination of endogenous modified nucleosides and deoxynucleosides in biological matrices. ISR allows to assess the robustness of the method and the stability of the analytes during storage and processing as well as the repeatability of the method over a long period of time. In the present study, 15 out of 133 urine samples from randomly selected patients were newly prepared and analyzed. The results obtained are presented in Table 6. It illustrates the determined percentage difference between the initially tested concentration and the concentration determined after repeated analysis. Obtained results met the EMA criteria according to which the calculated concentrations should be within 20% of their mean in 67% of the repeats. As it can be observed in Table 6, the newly prepared and determined analyte concentrations in the set of reanalyzed urine samples did not differ by more than 20% from the previously determined concentrations in more than 67% repeats. Three individual results deviated from the established criteria but only for single samples. For the compounds 2mG, 6mA, and 3mU, the determined concentrations differed from those previously calculated by 22.2%, 23.4%, and 34.6%, respectively. The rest of the determined concentrations for these analytes met the EMA standards. It can therefore be concluded that the determined incurred sample reanalysis parameter confirmed the robustness of the method as well as its repeatability over a longer period of time.

**Table 6:** Results of incurred sample reanalysis presenting the percent difference between the initially tested concentration and the concentration determined after repeated analysis

Sample/analyte	Pse [%]	U [%]	Ino [%]	3mU [%]	2dG [%]	2mG [%]	8OH2dG [%]	6mA [%]	2,2dMG [%]	5-MTA [%]	MTA [%]
P5	20.1	20.5	20.0	34.6	18.9	19.9	9.9	17.2	8.5	16.5	11.4
P15	1.4	13.3	5.2	13.7	9.8	19.5	18.8	14.6	15.0	19.2	15.3
P24	19.9	17.0	19.6	20.1	19.3	4.3	19.9	19.4	3.1	14.0	11.2
P26	12.3	17.7	14.5	18.3	10.6	3.9	18.9	16.0	8.2	19.4	12.2
P28	3.0	3.6	7.5	20.3	12.1	22.2	18.9	1.4	18.7	2.8	5.1
P38	14.7	20.0	17.5	19.0	13.1	14.5	5.1	16.5	5.6	19.7	13.8
P42	10.5	15.1	17.8	19.0	14.8	17.7	12.7	19.7	19.5	15.5	13.0
P43	11.9	5.9	8.5	18.7	19.3	11.3	19.8	18.2	16.6	2.7	11.3
P45	4.3	5.3	13.0	17.6	6.3	3.9	10.8	15.5	5.1	1.3	18.2
P46	7.5	10.3	4.3	19.1	9.6	15.0	15.6	20.5	1.3	5.5	6.4
P57	19.8	18.7	10.7	16.0	15.1	5.6	15.1	18.9	3.7	0.1	8.7
P67	16.8	19.4	4.9	19.2	17.1	4.6	16.3	14.3	0.8	11.3	3.4
P73	17.5	13.2	17.1	20.0	4.9	17.6	15.8	23.4	3.4	9.3	15.2
P74	19.9	19.9	17.4	16.5	19.2	9.5	17.6	19.8	5.0	16.0	19.1
P82	15.4	16.3	18.7	18.6	12.2	12.0	10.1	18.6	1.7	4.8	1.5

*Pse* pseudouridine, *U* uridine, *Ino* inosine, *3mU* N3-methyluridine, *2dG* 2-deoxyguanosine, *2mG* N2-methylguanosine, *8OH2dG* 8-hydroxy-2-deoxyguanosine, *6mA* N6-methyladenosine, *2,2dmG* N2,N2-dimethylguanosine, *8BrG* 8-bromoguanosine (*IS* internal standard), *5-MTA* 5-deoxy-5-methylthioadenosine, *MTA* 2-methylthioadenosine

## Conclusions

In the present study, a fast, simple, and accurate quantitative method for the simultaneous analysis of 11 nucleosides and deoxynucleosides from urine samples was developed and validated in terms of selectivity, linearity, accuracy, precision, recovery, matrix effect, and stability. There are not many procedures available where these two groups of compounds are analyzed simultaneously. The developed procedure is characterized by a particularly simple sample preparation procedure, including only centrifugation, mixing with a suitable solution, and evaporation steps. The method was successfully applied for the analysis of 133 urine samples obtained from 53 bladder cancer patients before and up to 12 months after tumor resection surgery to verify the potential correlation between changes of nucleoside and deoxynucleoside concentration and presence or excision of bladder tumor. Using a linear mixed-effect model strategy, two metabolites, 2-methylthioadenosine and inosine, were revealed to have the highest impact in terms of their level alterations before and 24 h after tumor resection. Some assumptions regarding the source of these observations were described. Unfortunately, the proper biochemical interpretation evaluating the role of these two metabolites is limited and requires further, extended analyses. However, we demonstrated that the method was reliable and easily applicable to a large set of samples within a relatively short time of analysis. Moreover, performed incurred sample reanalysis confirmed the robustness of the method. The newly developed method can be used for further studies on the importance of these metabolites in bladder cancer prognosis.

## Supplementary Information

Below is the link to the electronic supplementary material.

Supplementary file1 (PDF 1764 KB)
