# Supplementary material for: Targeted quantitative metabolomics with a linear mixed-effect model for analysis of urinary nucleosides and deoxynucleosides from bladder cancer patients before and after tumor resection
Source: Anal Bioanal Chem. 2023 Jul 18;415(22):5511–28. doi: 10.1007/s00216-023-04826-0 (PMC10444683; doi:10.1007/s00216-023-04826-0)
Supplement: Supplementary file 1 — Supplementary file1 (PDF 1764 KB) [file 216_2023_4826_MOESM1_ESM.pdf]

Table SM1. The characteristics of patients enrolled in the study.

No.	Age	Sex	BMI	TNM classification
1.	62	F	19.0	cT2
2.	74	M	24.8	cTa
3.	71	M	25.7	no information
4.	65	M	26.5	no information
5.	77	M	31.1	no information
6.	65	M	29.8	cTa
7.	81	M	24.2	cT1
8.	68	M	18.6	no information
9.	51	F	21.8	no information
10.	80	M	31.3	cT1
11.	73	M	36.0	cT0
12.	66	M	26.3	cT4
13.	80	M	23.3	cT2
14.	75	M	25.9	cT2
15.	70	M	23.8	cT2/T3
16.	73	M	25.1	cT2/T3
17.	73	M	23.3	cT2
18.	72	M	26.1	cT3bN3M1
19.	75	F	34.2	cTa/1
20.	77	M	27.4	cT1
21.	81	M	28.7	cT2
22.	77	M	24.2	cTa
23.	72	F	20.7	no information
24.	78	M	23.0	cTa/cT1
25.	67	M	25.8	cT1
26.	59	F	40.9	no information
27.	71	M	29.1	cTa
28.	83	M	24.3	cTa
29.	69	M	31.0	cT3bN1Mx
30.	65	F	15.6	cTa
31.	72	M	29.4	no information
32.	83	M	22.4	cT2
33.	71	F	30.5	no information
34.	63	M	29.3	cTa
35.	62	M	39.8	cT2
36.	78	M	23.1	cTa
37.	82	F	25.6	cT3
38.	61	M	33.6	cT1/T2
39.	82	F	32.9	cT1/T2
40.	75	M	26.1	cT1
41.	72	M	28.7	cT1/cT2
42.	68	M	32.5	no information
43.	78	F	26.3	cTa
44.	51	F	26.7	cT1/cT2
45.	35	F	22.0	cT1
46.	72	F	20.5	no information
47.	73	M	23.6	no information
48.	72	F	26.0	cTa
49.	74	F	21.3	no information
50.	75	M	28.3	cT4N+
51.	75	F	31.2	cT1
52.	64	M	27.8	no information
53.	76	F	23.4	cTa

Table legend: BMI- body mass index; F- female; M-male.

Table SM2. Chemical structures and molecular weight of modified nucleosides and deoxunuclesides.

Compound	Chemical structure	Molecular weight
Pseudouridine	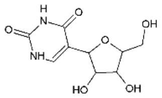	244.20
2-deoxyguanosine	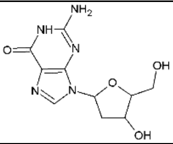	267.24
2-methylthioadenosine	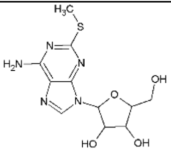	313.33
8-bromoguanosine	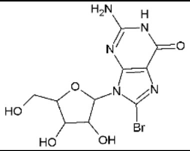	362.14
8-hydroxy-2-deoxyguanosine	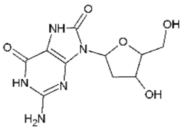	283.24
Inosine	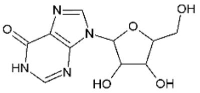	268.23
N2,N2- dimethylguanosine	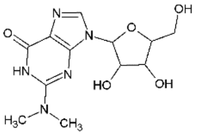	311.29
N2-methylguanosine	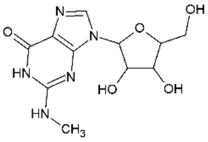	297.27
N3-methyluridine	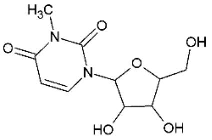	258.23
N6-methyladenosine	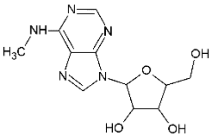	281.27
Uridine	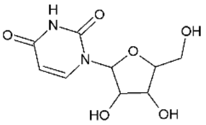	244.20

5-methylthioadenosine	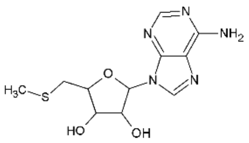	297.34

Chemical structures and molecular weight were obtained with the use of programme ACD Labs 12.0 (Advanced Chemistry Development Inc., Toronto, Canada).

Figure SM1. Product ion spectra of each modified nucleosides and deoxynucleosides. Above chosen quantifier and qualifier ions proposed fragments are presented.

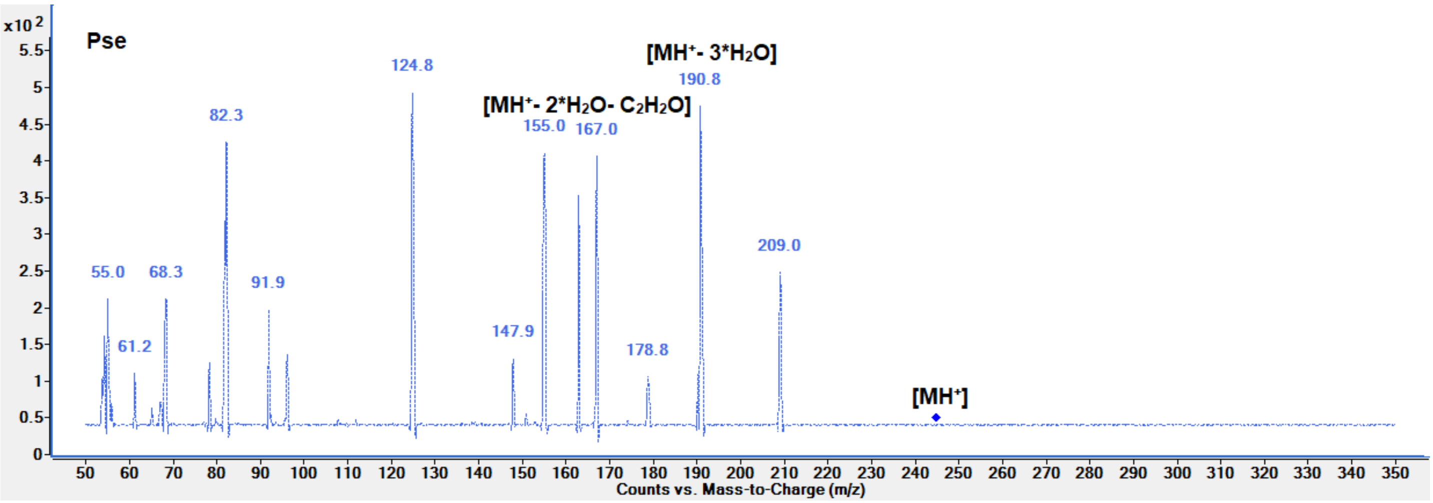

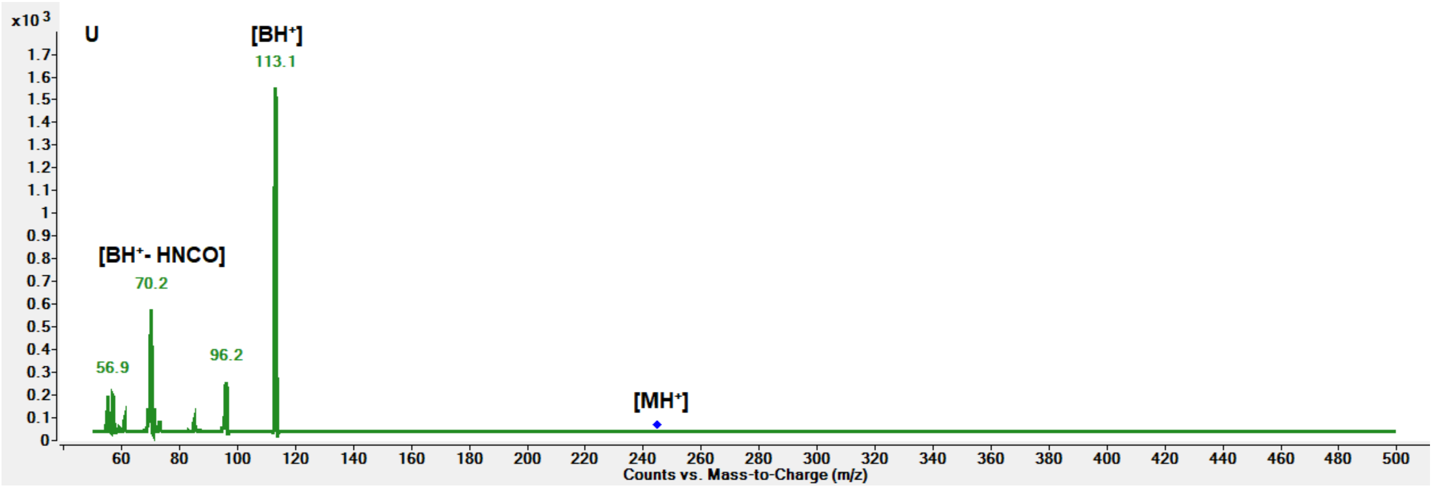

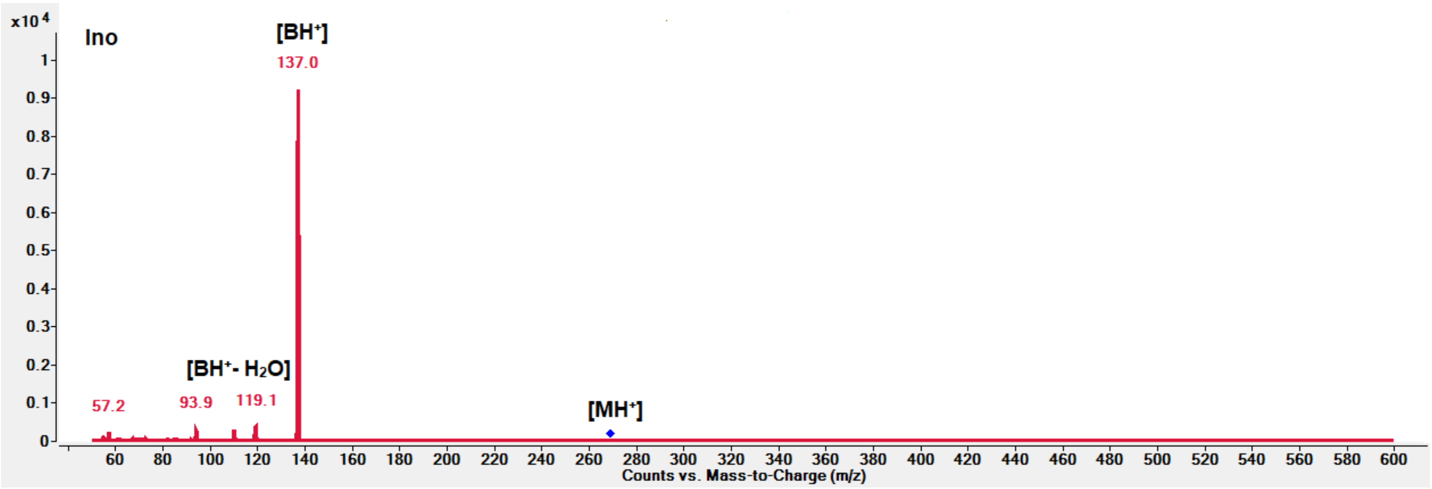

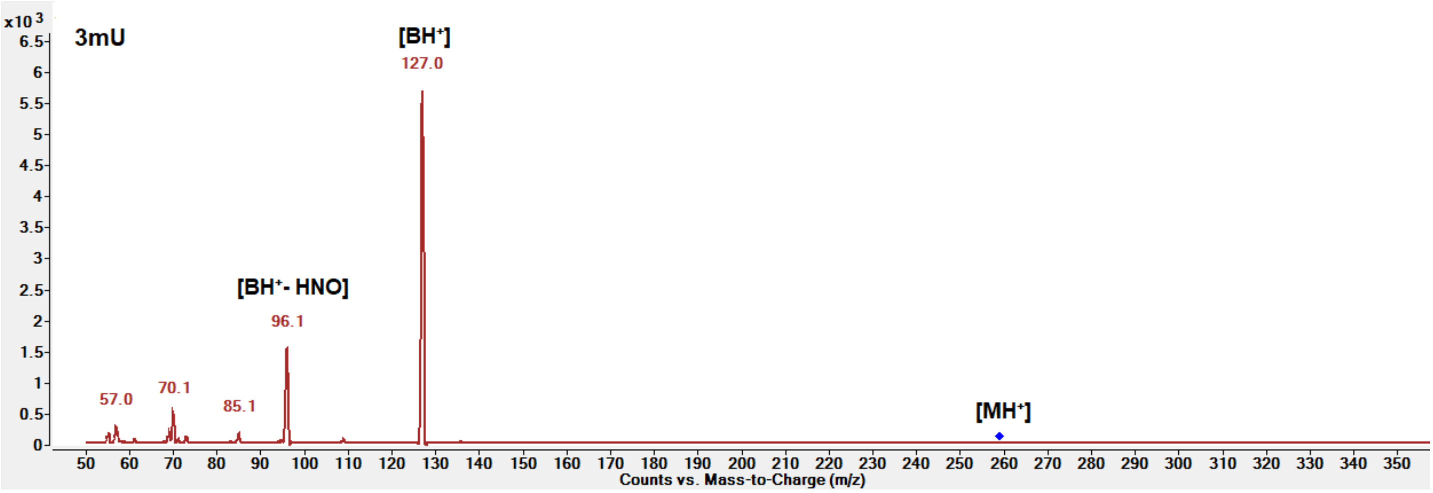
x10 3
3mU
[BH+]
127.0
[BH+- HNO]
96.1
57.0
70.1
85.1
[MH+]
Counts vs. Mass-to-Charge (m/z)

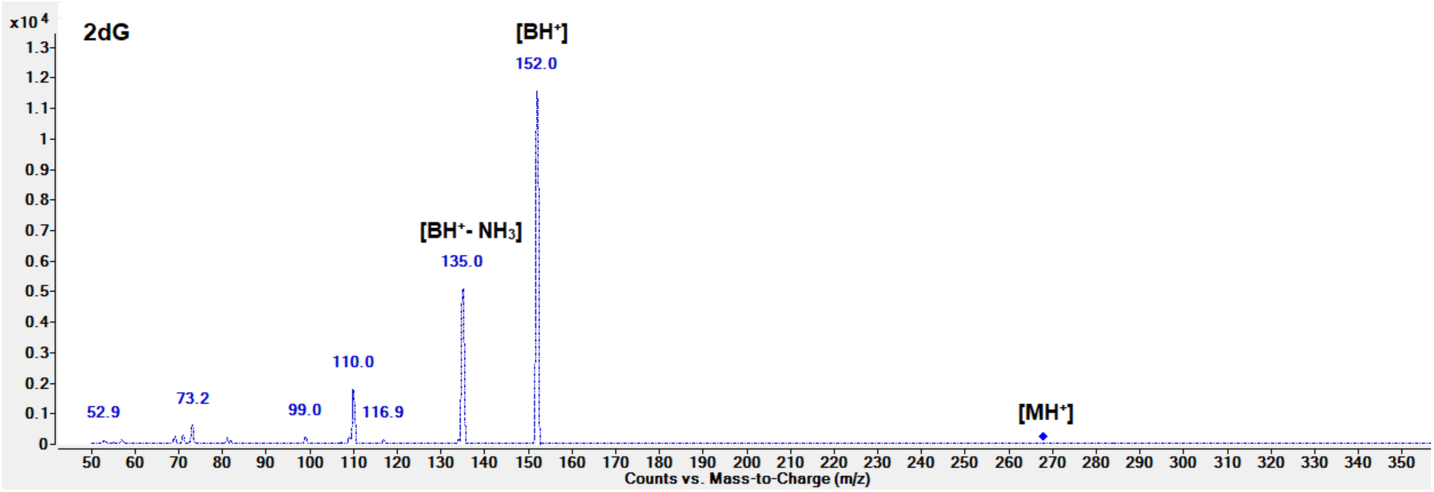
x10 4
2dG
[BH+]
152.0
[BH+- NH3]
135.0
110.0
52.9
73.2
99.0
116.9
[MH+]
Counts vs. Mass-to-Charge (m/z)

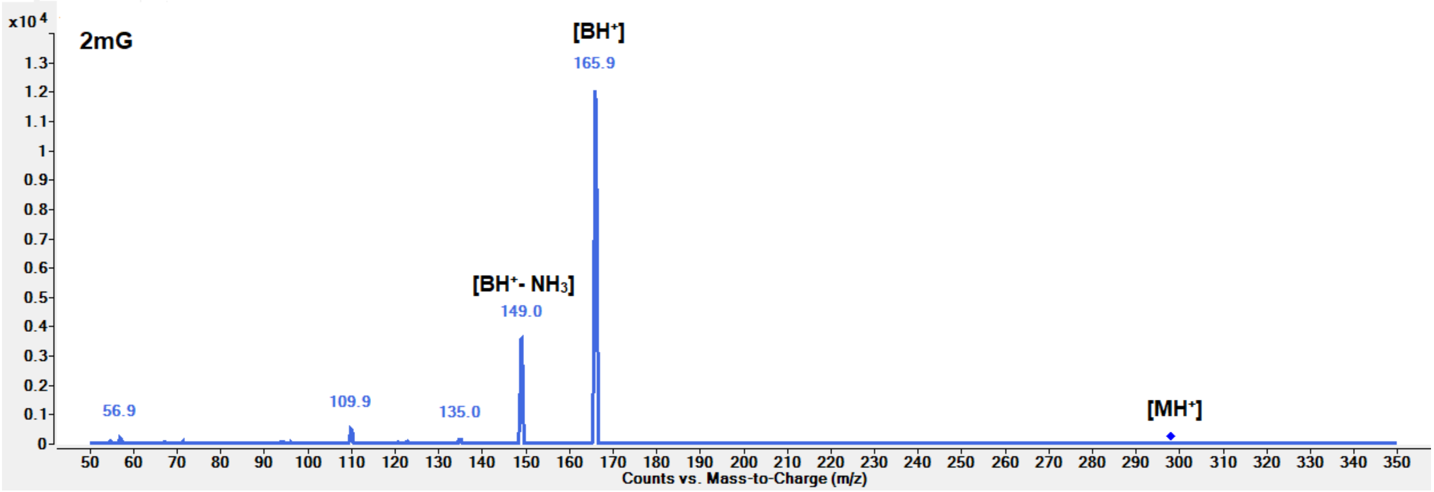
x10 4
2mG
[BH+]
165.9
[BH+- NH3]
149.0
56.9
109.9
135.0
[MH+]
Counts vs. Mass-to-Charge (m/z)

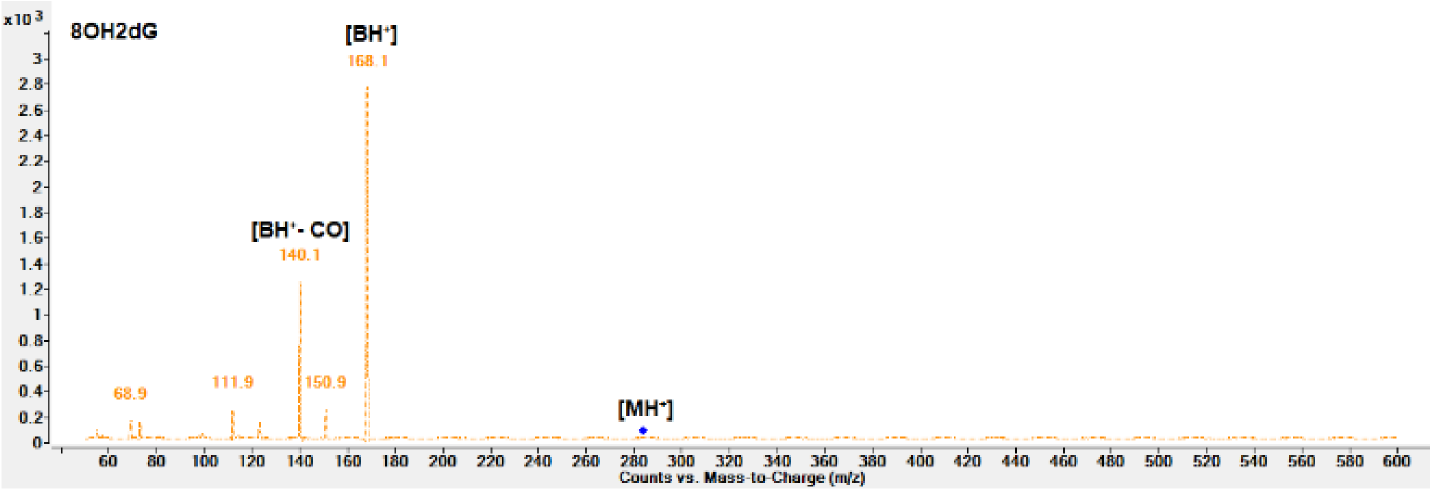

x10 3
8OH2dG
[BH⁺]
168.1
[BH⁺- CO]
140.1
68.9
111.9
150.9
[MH⁺]
Counts vs. Mass-to-Charge (m/z)

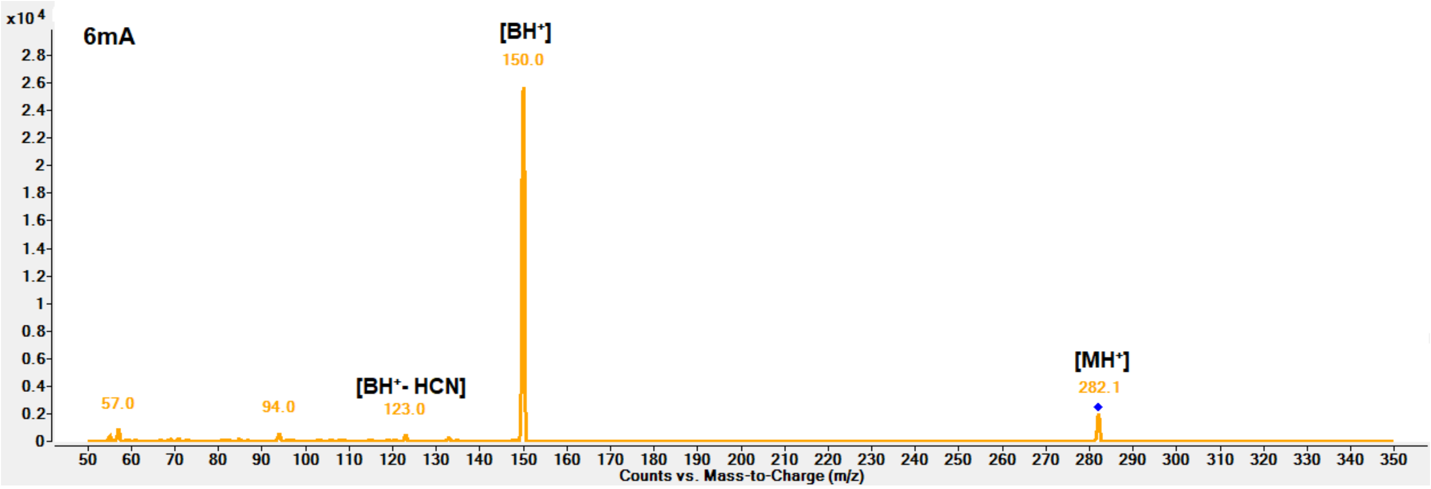

x10 4
6mA
[BH⁺]
150.0
[BH⁺- HCN]
123.0
57.0
94.0
[MH⁺]
282.1
Counts vs. Mass-to-Charge (m/z)

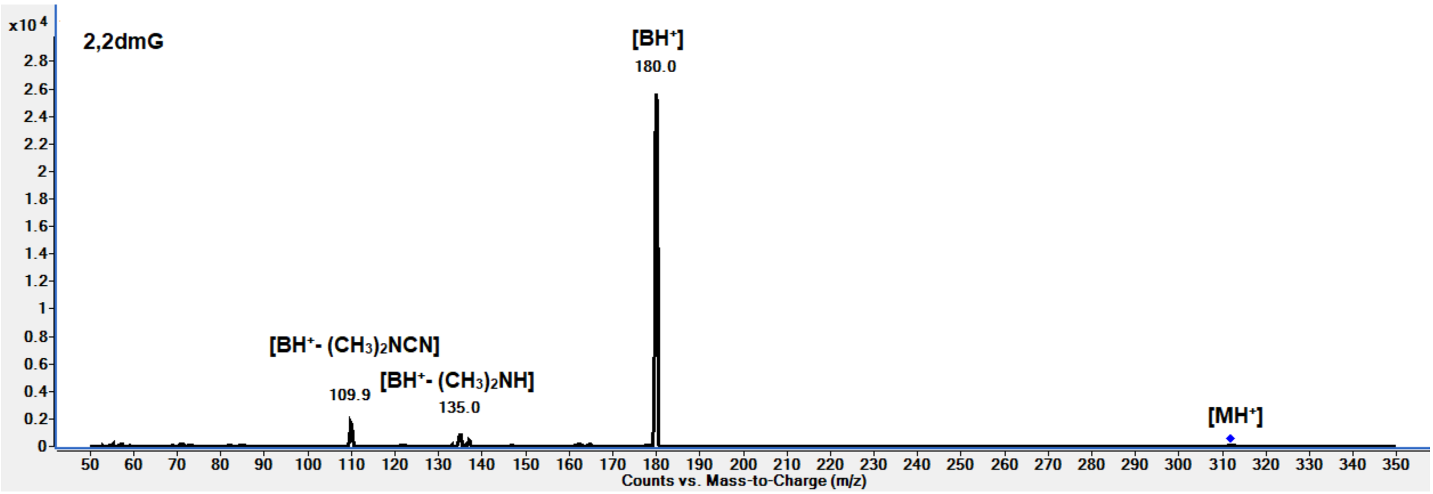

x10 4
2,2dmG
[BH⁺]
180.0
[BH⁺- (CH3)2NCN]
109.9
[BH⁺- (CH3)2NH]
135.0
[MH⁺]
Counts vs. Mass-to-Charge (m/z)

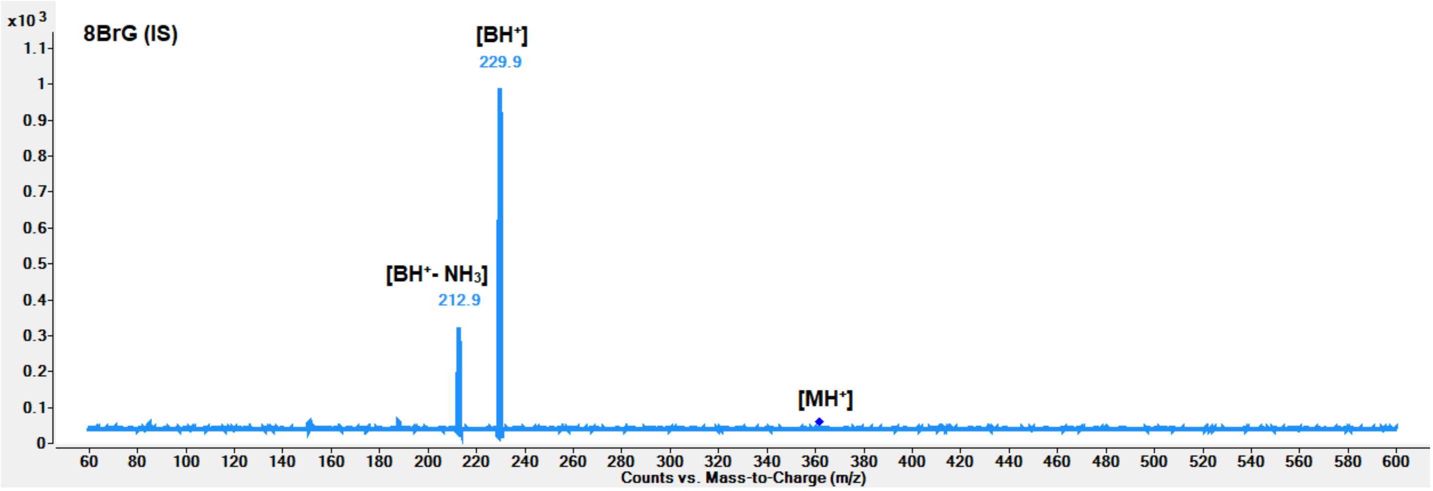
8BrG (IS)
x10 3
[BH⁺]
229.9
[BH⁺- NH3]
212.9
[MH⁺]
Counts vs. Mass-to-Charge (m/z)

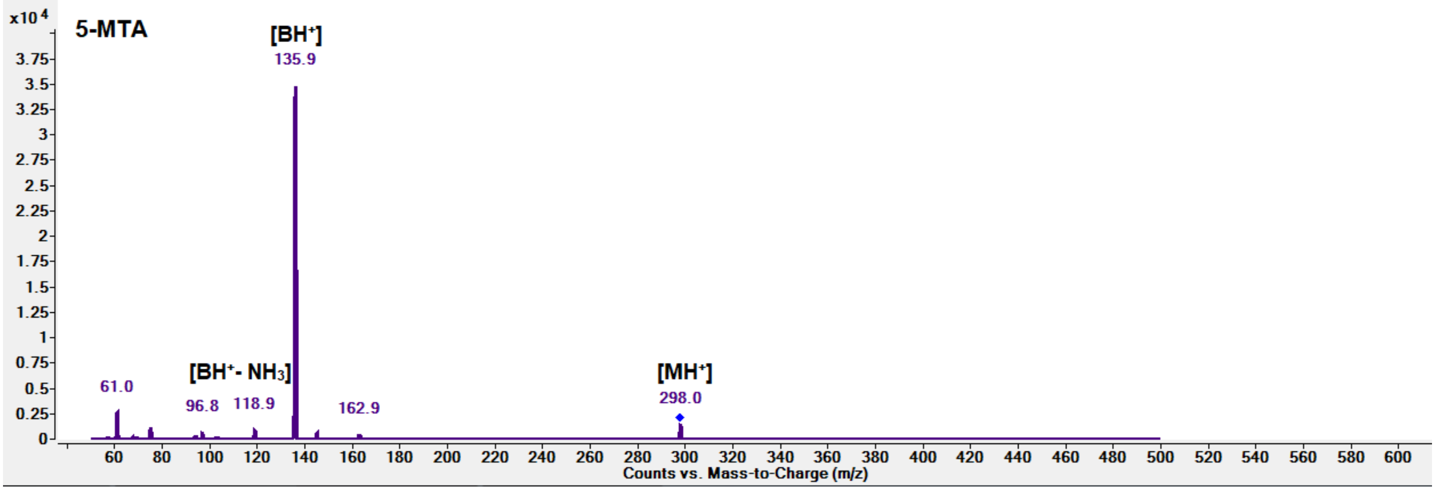
5-MTA
x10 4
[BH⁺]
135.9
61.0
[BH⁺- NH3]
96.8
118.9
162.9
[MH⁺]
298.0
Counts vs. Mass-to-Charge (m/z)

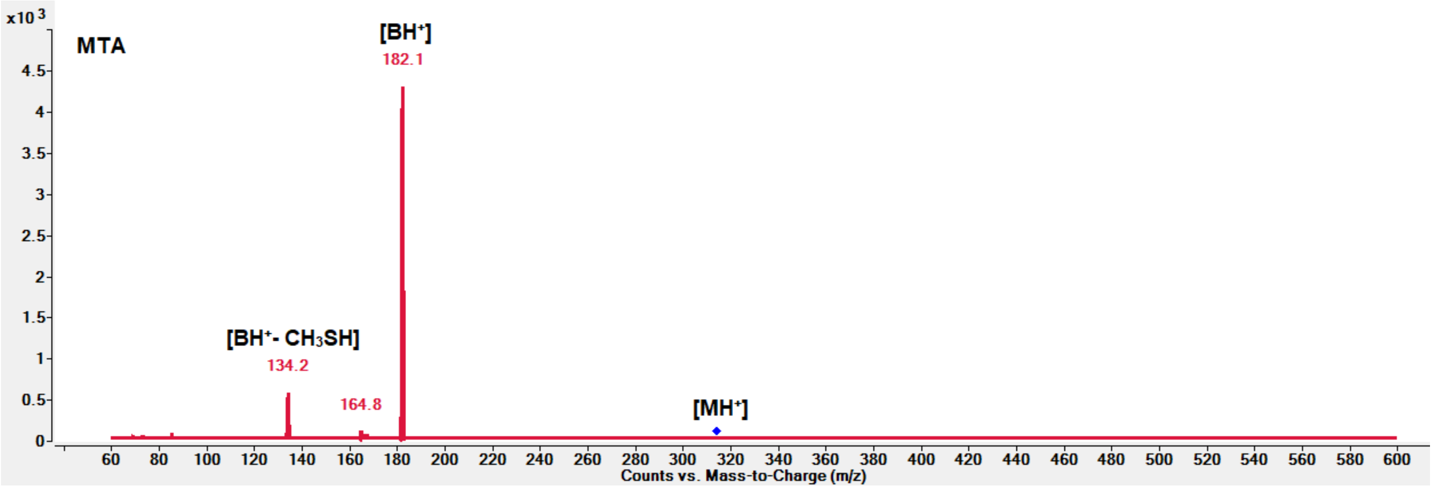
MTA
x10 3
[BH⁺]
182.1
[BH⁺- CH3SH]
134.2
164.8
[MH⁺]
Counts vs. Mass-to-Charge (m/z)

Figure legend*:* Pse- pseudouridine; U- uridine; Ino- inosine; 3mU- N3-methyluridine; 2dG- 2-deoxyguanosine; 2mG- N2-methylgoanosine; 8OH2dG- 8-hydroxy-2-deoxyguanosine; 6mA- N6-methyladenosine; 2,2dmG- N2,N2-dimethylguanosine; 8BrG- 8-bromoguanosine (IS- internal standard); 5-MTA- 5-deoxy-5-methylthioadenosine; MTA- 2-methylthioadenosine.

[MH^+] is the protonated molecule (precursor ion) while [BH^+] is the protonated base created by the loss of the neutral 2'-deoxyribose moiety (116u) in case of deoxynucleosides or ribose moiety (132u) in case of nucleosides. It is the characteristic fragmentation of nucleosides and deoxynucleosides. For every selected analyte, except Pse and 2,2dmG, [BH^+] was chosen as quantifier ion. The following fragmentation of the [BH^+] is caused by the ring opening and consequently loss of various molecules, depending on the structure of base. The most abundant fragment ions of [BH^+] were selected as qualifier ions.

Pseudouridine, in contrast to other nucleosides, has C-C bond between nucleobase and sugar moiety instead of C1' (sugar)- N1 (uracil) linkage. This cause difference in fragmentation pattern. Product ions are created through the loss of one, two or three water molecules as well as various fragments derived from both sugar and base moiety.

N2,N2-dimethylguanosine fragmentation pattern is typical as for other modified nucleosides. However, in presented study, in order to decrease the high concentration of 2,2-dmG in urine, less abundant ions, created from the fragmentation of protonated base, were chosen as product ions.

Suplementary Materials for
Targeted quantitative metabolomics with a linear mixed-effect model for analysis of urinary nucleosides and deoxynucleosides from bladder cancer patients before and after tumor resection

Małgorzata Artymowicz
Wiktoria Struck-Lewicka
Paweł Wiczling
Marcin Markuszewski
Michał J. Markuszewski
Danuta Siluk

2023-05-18

Contents

Setup **2**

Load data **2**

Exploratory graphical analisis **3**

Raw data grouped by metabolite 3

Correlation plots: 5

Data analysis **6**

Posterior predictive check **7**

Summary of paramters **7**

Conditional effects **13**

Visualise estimated paramters **14**

Hypothesis testing **15**

Variation in duresis **18**

Conclusions **18**

Session info **18**

Setup

Load packages:

```
library(dplyr)
library(ggplot2)
require(gridExtra)
library(brms)
library(GGally)
library(knitr)
library(tidybayes)
library(tidyr)

set.seed(10271998) ## not required but assures repeatable results
```

Load data

The raw data comprise urine concentration measurements [μM] of selected nucleosides, deoxynucleosides and creatinine. The data was collected at 7 time points (before, 24 h, about 1, 3, 6, 9, and 12 months after the transurethral resection of bladder tumor).

```
data_wide = read.csv('RawData/data.csv')

data_wide <- data_wide %>%
  mutate(CRE = CRE*1000)%>%
  mutate(CRE2 = CRE)
head(data_wide)
```

```
  Sample     ID    TimePointName TimePoint       Pse         U       Ino
1    P77  70166    przed TURT (1)         1  93.52751 0.5016063 0.6440434
2   P101  70166 24 h po TURT (2)         2  98.24309 0.6935635 1.5061076
3    P18  73260    przed TURT (1)         1 105.17025 0.2500165 0.1180694
4    P59  73260 24 h po TURT (2)         2  89.46857 0.2969947 0.2396231
5    P44 245601    przed TURT (1)         1  62.94275 0.0535473 0.3924358
6   P120 245601 24 h po TURT (2)         2 119.16007 0.5630087 0.5553789
       X3mU         X2dG      X2mG     X8OH2dG        X6mA   X22dmG     X5MTA
1 0.4848454 0.003008254 1.4678273 0.005769209 0.036421091 4.105970 0.1533563
2 0.3352376 0.004269248 1.8666644 0.005851973 0.030563392 5.065721 0.1790346
3 0.5096591 0.001518050 0.8891485 0.005994029 0.031611786 3.775939 0.1521225
4 0.3012004 0.001174943 0.7533241 0.005378283 0.037223485 4.942619 0.1036494
5 0.2554616 0.005360088 0.9280262 0.010323317 0.006394767 4.506682 0.1457223
6 0.6739679 0.005752024 1.8054542 0.007878822 0.279760491 6.300686 0.5691142
          MTA      CRE Age FOM1  BW     CRE2
1 0.000778936 1831.683  72    1  80 1831.683
2 0.000533899 4847.949  62    1 111 4847.949
3 0.000569433 3292.963  51    0  78 3292.963
4 0.000431037 3993.989  72    0  58 3993.989
5 0.000693714 3190.417  81    1  83 3190.417
6 0.000351730 3834.866  65    1  86 3834.866
```

The data was transformed to a long format. Three variables were added: i) creatinine normalized concentrations (DVCRE), (ii) logarithm of concentrations (logDV) and (iii) logarithm of creatinine normalized concentrations (logDVCRE):

```
data_long <- tidyr::gather(data_wide, MET, DV, Pse:CRE, factor_key=TRUE)

data_long <- data_long %>%
  mutate(logDV = log(DV)) %>%
  mutate(logDVCRE = log(DV/CRE2))%>%
  mutate(DVCRE = DV/CRE2)
```

Exploratory graphical analisis

Raw data grouped by metabolite

First plot displays concentrations. The second plot displays creatinine normalized concentrations.

```
new_labels = c("Pse", "U", "Ino", "3mU", "2dG", "2mG", "8OH2dG", "6mA","22dmG","5MTA","MTA", "CRE")
names(new_labels) <- c( "Pse","U","Ino","X3mU","X2dG","X2mG","X8OH2dG","X6mA","X22dmG","X5MTA","MTA","C[illegible]

p <- ggplot(subset(data_long, TimePoint<8))+
      geom_line(aes(x = TimePoint, y = DV, group = as.factor(ID), color="gray"))+
      geom_point(aes(x = TimePoint, y = DV, group = as.factor(ID), color="gray"))+
      geom_boxplot(aes(x = TimePoint, y = DV, fill=as.factor(TimePoint)),alpha=0.5) +
      scale_color_manual(values = "gray")+
        scale_y_continuous(trans='log10') +
        scale_x_continuous(breaks=seq(0,7,1)) +
      facet_wrap(.~as.factor(MET), scales="free", labeller = as_labeller(new_labels))+
      labs(title=element_blank(), x ="Time Points", y = "Concentrations, \u03BcM", color = "ID")+
      theme(legend.position="none")

print(p)
```

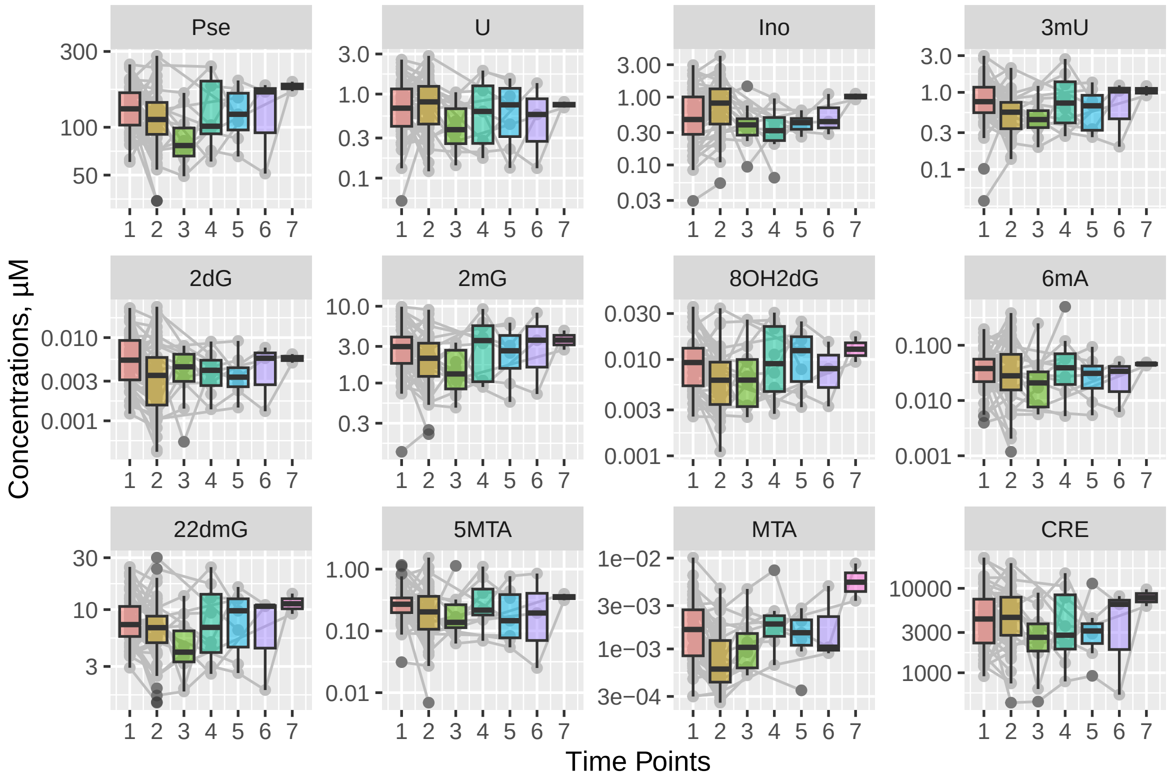


```
ggsave("Manuscript/RawData.png", plot=p,  width = 15*1.4, height = 15, units = "cm", dpi=300)

p <- ggplot(subset(data_long, TimePoint<8))+
      geom_line(aes(x = TimePoint, y = DVCRE, group = as.factor(ID), color="gray"))+
      geom_point(aes(x = TimePoint, y = DVCRE, group = as.factor(ID), color="gray"))+
      geom_boxplot(aes(x = TimePoint, y = DVCRE, fill=as.factor(TimePoint)),alpha=0.5) +
      scale_color_manual(values = "gray")+
         scale_y_continuous(trans='log10') +
        scale_x_continuous(breaks=seq(0,7,1)) +
      facet_wrap(.~as.factor(MET), scales="free", labeller = as_labeller(new_labels))+
      labs(title=element_blank(), x ="Time Points", y = "Creatinine-normalized Concentrations", color =
      theme(legend.position="none")

print(p)
```

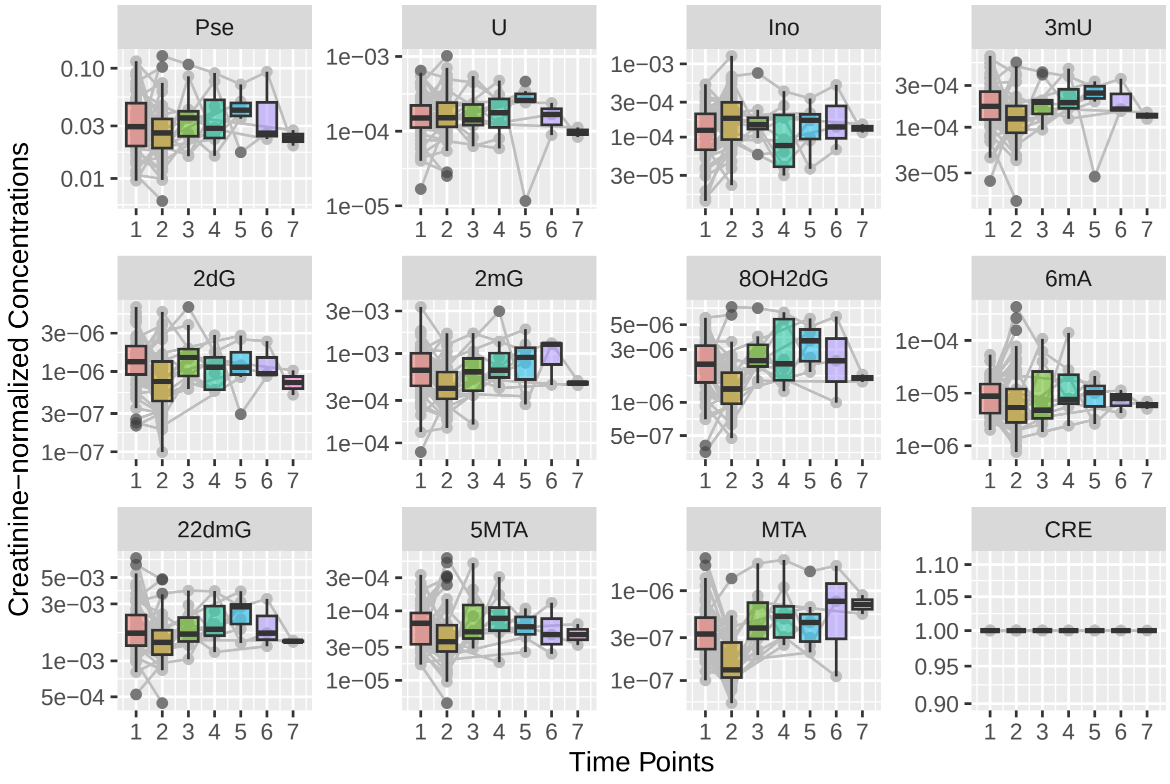

Correlation plots:

There is a high correlation between metabolite concentrations. It is likely due to the variation in diuresis (urine dilution).

```
data_wide_2 <- data_long %>%
  select(-c(CRE2, Sample, logDVCRE, DVCRE, DV)) %>%
  tidyr::pivot_wider(names_from = MET, values_from = logDV)

ggpairs(data_wide_2, columns = 7:(11+7),
        labeller = "label_parsed",
        upper = list(continuous = wrap("cor", size = 2)))+
theme(axis.text = element_text(size = 5))+
theme(strip.text.x = element_text(size = 5))+
theme(strip.text.y = element_text(size = 5))
```

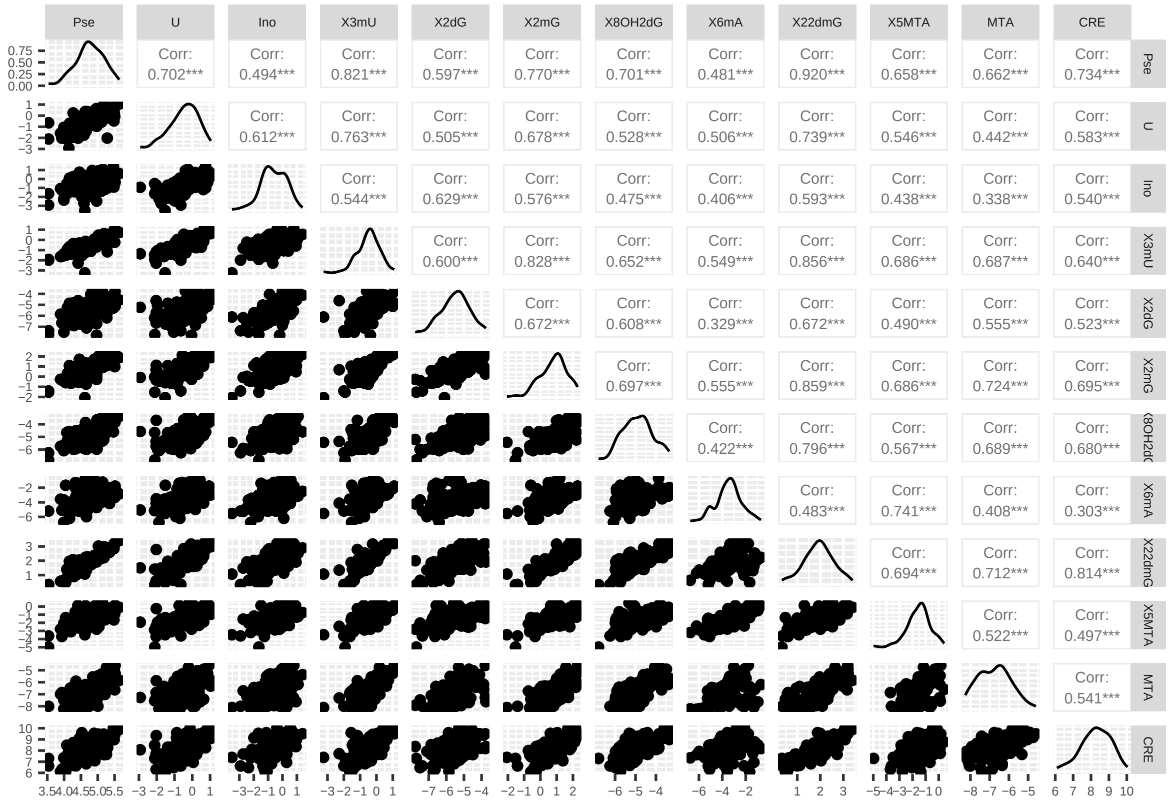

```r
rm(data_wide_2)
```

Data analysis

The data was analyzed using brms package. It fits the Bayesian generalized (non-)linear multivariate multilevel models using Stan. The default priors were used. The Time Points 3:7 were combined assuming no changes in urine concentrations for times > 2 weeks. The model assumes fixed effects of TimePoint, MET and the interaction of MET:TIMEPoint. The Sample and ID were modeled as random effects. The variance for between patient variability was different for each MET and correlated. The variance for within subject variability was also assumed to be different for each MET.

```r
data_long$TimePoint = as.factor(data_long$TimePoint)

levels(data_long$TimePoint) <- c(1,2,3,3,3,3,3)

data_long$Sample = as.factor(data_long$Sample)

# get_prior(bf(
#     logDV ~ TimePoint + MET + MET:TimePoint + (1+MET|ID) + (1|Sample),
#     sigma ~ MET),
#     family = student,
#     data = data_long)
```

Fit the model:

```r
fit <- brm(bf(
              logDV ~ TimePoint + MET + MET:TimePoint + (1+MET|ID) + (1|Sample),
              sigma ~ MET),
  family = student,
  data = data_long,
  chains = 4,
  cores = 4)

saveRDS(fit, file = "fit.RDS")
```

Load saved data.

```r
fit <- readRDS(file = "fit.RDS")
```

Posterior predictive check

The agreement between the data and simulation confirms that model predicts the data well.

```r
pp_check(fit, ndraws = 100)
```

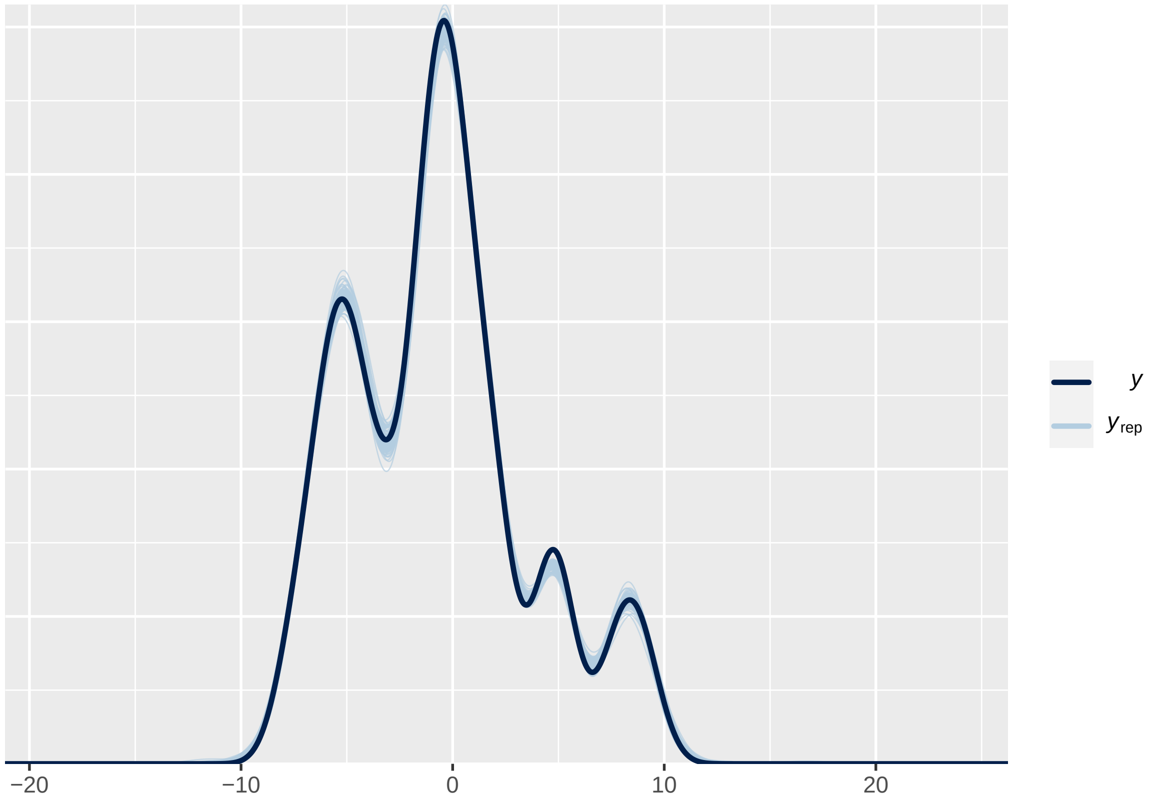

Summary of paramters

The marginal posterior distribution of each parameter. Posterior mean, standard error, 95% credible interval and Rhat statistic for each parameter of model.

```
summary(fit)
```

```
 Family: student
  Links: mu = identity; sigma = log; nu = identity
Formula: logDV ~ TimePoint + MET + MET:TimePoint + (1 + MET | ID) + (1 | Sample)
         sigma ~ MET
   Data: data_long (Number of observations: 1594)
  Draws: 4 chains, each with iter = 2000; warmup = 1000; thin = 1;
         total post-warmup draws = 4000

Group-Level Effects:
~ID (Number of levels: 53)
                          Estimate Est.Error l-95% CI u-95% CI Rhat Bulk_ESS
sd(Intercept)                 0.25      0.06     0.14     0.36 1.01     1180
sd(METU)                      0.35      0.05     0.27     0.45 1.00     2138
sd(METIno)                    0.59      0.07     0.47     0.73 1.00     2190
sd(METX3mU)                   0.10      0.03     0.04     0.15 1.00     1106
sd(METX2dG)                   0.39      0.06     0.29     0.51 1.00     2740
sd(METX2mG)                   0.37      0.04     0.29     0.46 1.00     2360
sd(METX8OH2dG)                0.44      0.05     0.36     0.55 1.00     2217
sd(METX6mA)                   0.36      0.11     0.14     0.58 1.00     1447
sd(METX22dmG)                 0.14      0.02     0.09     0.19 1.00     1240
sd(METX5MTA)                  0.19      0.07     0.03     0.33 1.00     1477
sd(METMTA)                    0.42      0.05     0.33     0.54 1.00     2813
sd(METCRE)                    0.32      0.08     0.15     0.47 1.00     1014
cor(Intercept,METU)           0.23      0.19    -0.15     0.57 1.00     1068
cor(Intercept,METIno)         0.11      0.19    -0.25     0.47 1.00      841
cor(METU,METIno)              0.39      0.13     0.11     0.62 1.00     1522
cor(Intercept,METX3mU)        0.46      0.21     0.01     0.79 1.00     1985
cor(METU,METX3mU)             0.33      0.19    -0.10     0.65 1.00     2974
cor(METIno,METX3mU)          -0.01      0.19    -0.40     0.36 1.00     3995
cor(Intercept,METX2dG)        0.14      0.19    -0.23     0.50 1.00     1272
cor(METU,METX2dG)             0.26      0.16    -0.07     0.55 1.00     2740
cor(METIno,METX2dG)           0.70      0.11     0.45     0.87 1.00     3149
cor(METX3mU,METX2dG)          0.01      0.20    -0.40     0.40 1.00     2530
cor(Intercept,METX2mG)        0.43      0.17     0.07     0.73 1.00      936
cor(METU,METX2mG)             0.17      0.15    -0.12     0.44 1.00     2938
cor(METIno,METX2mG)           0.17      0.14    -0.11     0.43 1.00     2208
cor(METX3mU,METX2mG)          0.33      0.19    -0.10     0.66 1.00      973
cor(METX2dG,METX2mG)          0.32      0.15     0.02     0.60 1.00     2105
cor(Intercept,METX8OH2dG)     0.06      0.19    -0.32     0.43 1.00      680
cor(METU,METX8OH2dG)          0.04      0.15    -0.26     0.32 1.00     2342
cor(METIno,METX8OH2dG)        0.08      0.14    -0.21     0.34 1.00     2756
cor(METX3mU,METX8OH2dG)      -0.03      0.19    -0.41     0.34 1.00      643
cor(METX2dG,METX8OH2dG)       0.11      0.15    -0.21     0.40 1.00     1561
cor(METX2mG,METX8OH2dG)       0.07      0.14    -0.22     0.33 1.00     2287
cor(Intercept,METX6mA)        0.24      0.21    -0.18     0.63 1.00     1855
cor(METU,METX6mA)             0.18      0.20    -0.21     0.55 1.00     3652
cor(METIno,METX6mA)           0.20      0.19    -0.19     0.54 1.00     4693
cor(METX3mU,METX6mA)          0.22      0.22    -0.25     0.60 1.00     1669
cor(METX2dG,METX6mA)          0.05      0.22    -0.37     0.47 1.00     3271
cor(METX2mG,METX6mA)          0.44      0.18     0.06     0.74 1.00     4382
cor(METX8OH2dG,METX6mA)       0.05      0.20    -0.35     0.42 1.00     4842
```

```
cor(Intercept,METX22dmG)       0.60      0.15     0.27     0.84 1.00     1628
cor(METU,METX22dmG)            0.25      0.16    -0.09     0.54 1.00     2920
cor(METIno,METX22dmG)          0.13      0.15    -0.18     0.40 1.00     3818
cor(METX3mU,METX22dmG)         0.64      0.18     0.21     0.88 1.01     1287
cor(METX2dG,METX22dmG)         0.22      0.16    -0.11     0.50 1.00     3256
cor(METX2mG,METX22dmG)         0.42      0.14     0.12     0.68 1.00     2161
cor(METX8OH2dG,METX22dmG)      0.30      0.14     0.00     0.57 1.00     2986
cor(METX6mA,METX22dmG)         0.15      0.20    -0.26     0.52 1.00     2851
cor(Intercept,METX5MTA)        0.29      0.23    -0.18     0.69 1.00     2981
cor(METU,METX5MTA)             0.14      0.22    -0.30     0.55 1.00     4625
cor(METIno,METX5MTA)           0.18      0.21    -0.26     0.56 1.00     4847
cor(METX3mU,METX5MTA)          0.26      0.23    -0.22     0.67 1.00     2435
cor(METX2dG,METX5MTA)          0.11      0.22    -0.34     0.54 1.00     5416
cor(METX2mG,METX5MTA)          0.36      0.21    -0.11     0.73 1.00     3631
cor(METX8OH2dG,METX5MTA)       0.05      0.22    -0.39     0.46 1.00     5285
cor(METX6mA,METX5MTA)          0.30      0.24    -0.21     0.70 1.00     2985
cor(METX22dmG,METX5MTA)        0.33      0.22    -0.14     0.70 1.00     3727
cor(Intercept,METMTA)          0.33      0.18    -0.05     0.65 1.00      976
cor(METU,METMTA)              -0.00      0.15    -0.30     0.28 1.00     3770
cor(METIno,METMTA)             0.15      0.14    -0.14     0.41 1.00     4058
cor(METX3mU,METMTA)            0.17      0.20    -0.24     0.52 1.00     1256
cor(METX2dG,METMTA)            0.16      0.16    -0.16     0.46 1.00     2441
cor(METX2mG,METMTA)            0.33      0.14     0.03     0.58 1.00     3416
cor(METX8OH2dG,METMTA)         0.17      0.14    -0.11     0.43 1.00     4458
cor(METX6mA,METMTA)            0.29      0.19    -0.11     0.63 1.00     1778
cor(METX22dmG,METMTA)          0.30      0.15    -0.01     0.57 1.00     2587
cor(METX5MTA,METMTA)           0.11      0.22    -0.33     0.52 1.00     1884
cor(Intercept,METCRE)          0.29      0.20    -0.11     0.66 1.00     1471
cor(METU,METCRE)              -0.01      0.18    -0.35     0.34 1.00     3409
cor(METIno,METCRE)             0.04      0.17    -0.29     0.37 1.00     3978
cor(METX3mU,METCRE)            0.27      0.22    -0.21     0.65 1.00     1483
cor(METX2dG,METCRE)            0.10      0.18    -0.26     0.46 1.00     2953
cor(METX2mG,METCRE)            0.30      0.17    -0.05     0.62 1.00     3689
cor(METX8OH2dG,METCRE)         0.36      0.17     0.01     0.65 1.00     3743
cor(METX6mA,METCRE)            0.05      0.21    -0.37     0.46 1.00     2977
cor(METX22dmG,METCRE)          0.50      0.17     0.14     0.77 1.00     2188
cor(METX5MTA,METCRE)           0.22      0.23    -0.26     0.63 1.00     2506
cor(METMTA,METCRE)             0.21      0.17    -0.15     0.53 1.00     3186
                           Tail_ESS
sd(Intercept)                  1685
sd(METU)                       3113
sd(METIno)                     2923
sd(METX3mU)                     844
sd(METX2dG)                    3084
sd(METX2mG)                    2847
sd(METX8OH2dG)                 2887
sd(METX6mA)                    1406
sd(METX22dmG)                  1038
sd(METX5MTA)                   1419
sd(METMTA)                     2620
sd(METCRE)                     1375
cor(Intercept,METU)            1756
cor(Intercept,METIno)          1297
cor(METU,METIno)               2708
```

cor(Intercept,METX3mU)	1975
cor(METU,METX3mU)	2109
cor(METIno,METX3mU)	2965
cor(Intercept,METX2dG)	1965
cor(METU,METX2dG)	3426
cor(METIno,METX2dG)	3147
cor(METX3mU,METX2dG)	2507
cor(Intercept,METX2mG)	1761
cor(METU,METX2mG)	3315
cor(METIno,METX2mG)	3102
cor(METX3mU,METX2mG)	1506
cor(METX2dG,METX2mG)	3244
cor(Intercept,METX8OH2dG)	1204
cor(METU,METX8OH2dG)	2673
cor(METIno,METX8OH2dG)	2733
cor(METX3mU,METX8OH2dG)	1714
cor(METX2dG,METX8OH2dG)	2305
cor(METX2mG,METX8OH2dG)	2771
cor(Intercept,METX6mA)	2968
cor(METU,METX6mA)	3411
cor(METIno,METX6mA)	3170
cor(METX3mU,METX6mA)	2356
cor(METX2dG,METX6mA)	3194
cor(METX2mG,METX6mA)	3014
cor(METX8OH2dG,METX6mA)	3303
cor(Intercept,METX22dmG)	2531
cor(METU,METX22dmG)	2514
cor(METIno,METX22dmG)	3419
cor(METX3mU,METX22dmG)	895
cor(METX2dG,METX22dmG)	3260
cor(METX2mG,METX22dmG)	2869
cor(METX8OH2dG,METX22dmG)	3445
cor(METX6mA,METX22dmG)	2984
cor(Intercept,METX5MTA)	3495
cor(METU,METX5MTA)	2678
cor(METIno,METX5MTA)	3020
cor(METX3mU,METX5MTA)	2943
cor(METX2dG,METX5MTA)	3577
cor(METX2mG,METX5MTA)	2550
cor(METX8OH2dG,METX5MTA)	3181
cor(METX6mA,METX5MTA)	3160
cor(METX22dmG,METX5MTA)	3136
cor(Intercept,METMTA)	1350
cor(METU,METMTA)	3556
cor(METIno,METMTA)	3244
cor(METX3mU,METMTA)	2335
cor(METX2dG,METMTA)	3114
cor(METX2mG,METMTA)	3322
cor(METX8OH2dG,METMTA)	3459
cor(METX6mA,METMTA)	2801
cor(METX22dmG,METMTA)	3382
cor(METX5MTA,METMTA)	2997
cor(Intercept,METCRE)	2676
cor(METU,METCRE)	3007

cor(METIno,METCRE)	3531
cor(METX3mU,METCRE)	2101
cor(METX2dG,METCRE)	3500
cor(METX2mG,METCRE)	3351
cor(METX8OH2dG,METCRE)	3202
cor(METX6mA,METCRE)	3342
cor(METX22dmG,METCRE)	2230
cor(METX5MTA,METCRE)	3096
cor(METMTA,METCRE)	3029

~Sample (Number of levels: 133)

	Estimate	Est.Error	l-95% CI	u-95% CI	Rhat	Bulk_ESS	Tail_ESS
sd(Intercept)	0.45	0.03	0.39	0.52	1.00	1845	2759

Population-Level Effects:

	Estimate	Est.Error	l-95% CI	u-95% CI	Rhat	Bulk_ESS
Intercept	4.87	0.07	4.72	5.02	1.00	2319
sigma_Intercept	-1.96	0.14	-2.23	-1.69	1.00	946
TimePoint2	-0.15	0.10	-0.33	0.04	1.00	2118
TimePoint3	-0.17	0.12	-0.40	0.06	1.00	1755
METU	-5.22	0.07	-5.35	-5.09	1.00	2575
METIno	-5.62	0.10	-5.81	-5.42	1.00	2421
METX3mU	-5.08	0.03	-5.15	-5.02	1.00	2599
METX2dG	-10.13	0.08	-10.30	-9.97	1.00	3388
METX2mG	-3.84	0.07	-3.97	-3.72	1.00	3090
METX8OH2dG	-9.56	0.07	-9.69	-9.42	1.00	2898
METX6mA	-8.21	0.11	-8.43	-8.00	1.00	5015
METX22dmG	-2.82	0.03	-2.89	-2.76	1.00	2285
METX5MTA	-6.17	0.08	-6.32	-6.02	1.00	3888
METMTA	-11.32	0.07	-11.46	-11.17	1.00	3177
METCRE	3.53	0.08	3.37	3.68	1.00	3621
TimePoint2:METU	0.17	0.06	0.05	0.29	1.00	3354
TimePoint3:METU	-0.05	0.09	-0.22	0.12	1.00	3189
TimePoint2:METIno	0.55	0.08	0.40	0.70	1.00	4019
TimePoint3:METIno	0.03	0.10	-0.18	0.22	1.00	4121
TimePoint2:METX3mU	-0.22	0.05	-0.32	-0.13	1.00	2660
TimePoint3:METX3mU	-0.05	0.06	-0.17	0.06	1.00	2474
TimePoint2:METX2dG	-0.30	0.09	-0.49	-0.12	1.00	4063
TimePoint3:METX2dG	-0.19	0.11	-0.42	0.04	1.00	3826
TimePoint2:METX2mG	-0.25	0.06	-0.37	-0.13	1.00	2975
TimePoint3:METX2mG	-0.03	0.08	-0.18	0.12	1.00	3062
TimePoint2:METX8OH2dG	-0.26	0.05	-0.36	-0.16	1.00	2635
TimePoint3:METX8OH2dG	0.01	0.07	-0.12	0.14	1.00	2734
TimePoint2:METX6mA	-0.29	0.16	-0.59	0.01	1.00	5699
TimePoint3:METX6mA	-0.26	0.17	-0.60	0.07	1.00	4938
TimePoint2:METX22dmG	-0.06	0.04	-0.14	0.03	1.00	2289
TimePoint3:METX22dmG	-0.03	0.06	-0.14	0.08	1.00	2289
TimePoint2:METX5MTA	-0.34	0.12	-0.56	-0.12	1.00	4257
TimePoint3:METX5MTA	-0.16	0.13	-0.42	0.09	1.00	4022
TimePoint2:METMTA	-0.68	0.07	-0.82	-0.55	1.00	3379
TimePoint3:METMTA	0.03	0.09	-0.13	0.20	1.00	3495
TimePoint2:METCRE	0.14	0.09	-0.02	0.31	1.00	3924
TimePoint3:METCRE	-0.13	0.12	-0.36	0.10	1.00	3827
sigma_METU	0.30	0.18	-0.05	0.65	1.00	1447

```
sigma_METIno            0.54      0.17     0.21     0.89 1.00     1318
sigma_METX3mU          -0.22      0.18    -0.58     0.13 1.00     1586
sigma_METX2dG           0.78      0.17     0.44     1.12 1.00     1336
sigma_METX2mG           0.13      0.19    -0.24     0.50 1.00     1133
sigma_METX8OH2dG       -0.20      0.19    -0.57     0.18 1.00     1244
sigma_METX6mA           1.36      0.17     1.02     1.69 1.01     1124
sigma_METX22dmG        -0.63      0.20    -1.06    -0.25 1.00     1386
sigma_METX5MTA          1.04      0.16     0.72     1.36 1.00     1324
sigma_METMTA            0.34      0.18    -0.02     0.68 1.00     1414
sigma_METCRE            0.75      0.17     0.41     1.09 1.00     1553
                      Tail_ESS
Intercept                 2730
sigma_Intercept           1685
TimePoint2                2719
TimePoint3                2306
METU                      2745
METIno                    2715
METX3mU                   3006
METX2dG                   3061
METX2mG                   2881
METX8OH2dG                2862
METX6mA                   3359
METX22dmG                 3043
METX5MTA                  2917
METMTA                    3208
METCRE                    2816
TimePoint2:METU           3377
TimePoint3:METU           3339
TimePoint2:METIno         3186
TimePoint3:METIno         3248
TimePoint2:METX3mU        3011
TimePoint3:METX3mU        3390
TimePoint2:METX2dG        3070
TimePoint3:METX2dG        3466
TimePoint2:METX2mG        3215
TimePoint3:METX2mG        3359
TimePoint2:METX8OH2dG     3111
TimePoint3:METX8OH2dG     3198
TimePoint2:METX6mA        3724
TimePoint3:METX6mA        3061
TimePoint2:METX22dmG      3169
TimePoint3:METX22dmG      2828
TimePoint2:METX5MTA       2920
TimePoint3:METX5MTA       3335
TimePoint2:METMTA         3370
TimePoint3:METMTA         3067
TimePoint2:METCRE         3199
TimePoint3:METCRE         3403
sigma_METU                2217
sigma_METIno              1967
sigma_METX3mU             2241
sigma_METX2dG             2436
sigma_METX2mG             1772
sigma_METX8OH2dG          2674
```

```
sigma_METX6mA              2199
sigma_METX22dmG            2330
sigma_METX5MTA             1952
sigma_METMTA               2254
sigma_METCRE               2635

Family Specific Parameters:
   Estimate Est.Error l-95% CI u-95% CI Rhat Bulk_ESS Tail_ESS
nu     2.68      0.29     2.19     3.28 1.00     2815     3139

Draws were sampled using sampling(NUTS). For each parameter, Bulk_ESS
and Tail_ESS are effective sample size measures, and Rhat is the potential
scale reduction factor on split chains (at convergence, Rhat = 1).
```

Conditional effects

The following graphs display predicted concentrations for a typical subject and typical diuresis.

```
plot(conditional_effects(fit, effects="TimePoint:MET"))[[1]]+
      facet_wrap(.~ MET, scales="free", labeller = as_labeller(new_labels))
```

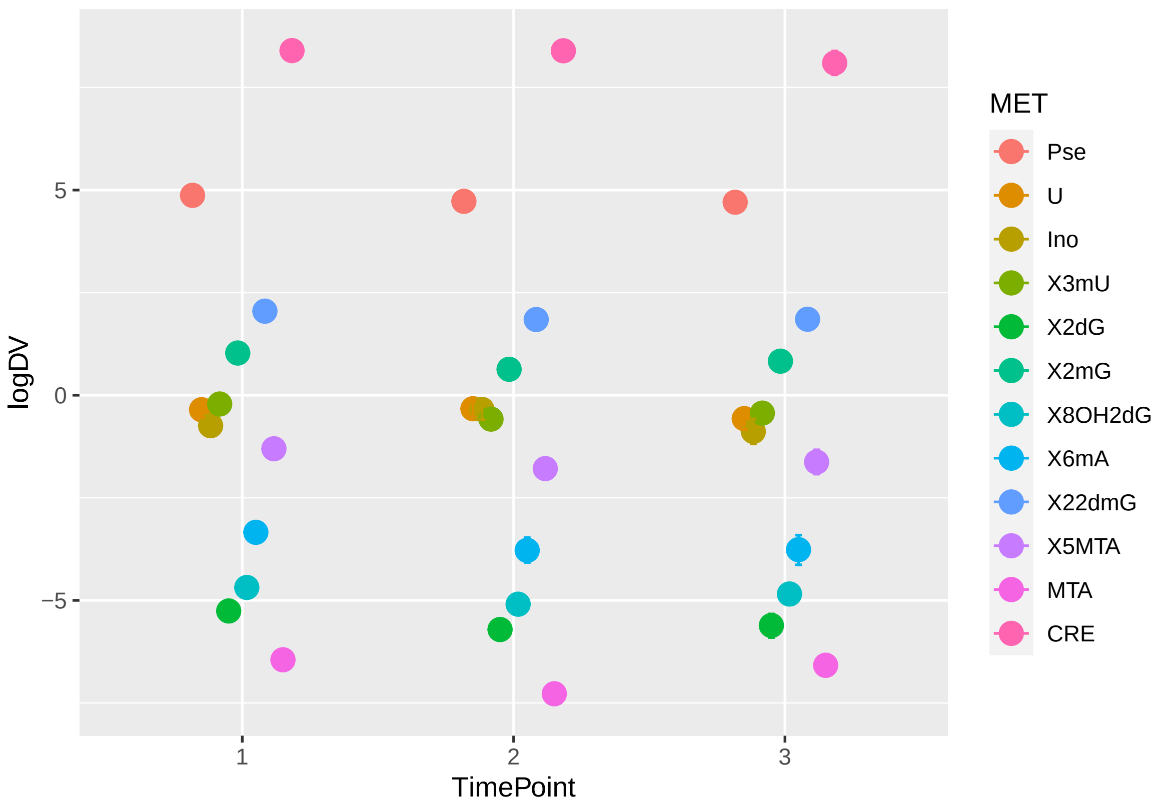

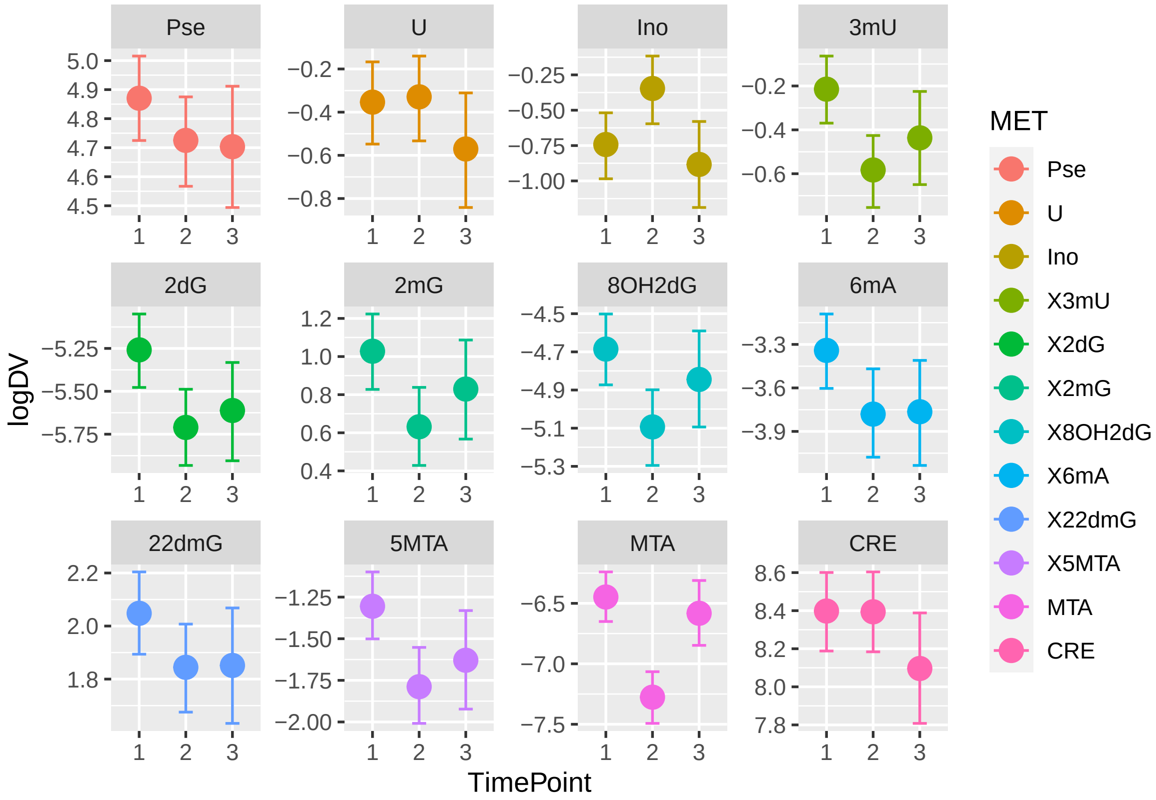

Visualise estimated paramters

The effect of intervention is represented as a difference in an average MET concentration (for a typical subject and typical diuresis) between $TimePoint$ 2 (or 3) and $TimePoint$ 1. The solid line corresponds to no difference between TimePoints. The broken lines shows $\pm$ 0.223 difference corresponding to (0.8-1.25) fold change.

```
new_labels = c("Pse", "U", "Ino", "3mU", "2dG", "2mG", "8OH2dG", "6mA","22dmG","5MTA","MTA", "CRE")


p1<-mcmc_plot(fit, variable = "^b_TimePoint2", regex = TRUE, transformation=exp)+
 geom_vline(xintercept = c(0.8,1.25), linetype = 3)+
  geom_vline(xintercept = c(1), linetype = 1) +
  xlim(0.4,2.1)+
  xlab("Fold change from baseline")+
  ggtitle("24 h post surgery")+
  scale_y_discrete(labels = rev(new_labels), limits = rev)

p2<-mcmc_plot(fit, variable = "^b_TimePoint3", regex = TRUE, transformation=exp)+
 geom_vline(xintercept = c(0.8,1.25), linetype = 3)+
geom_vline(xintercept = c(1), linetype = 1) +
  xlim(0.4,2.1)+
  xlab("Fold change from baseline")+
  ggtitle("> 2 weeks post surgery")+
  scale_y_discrete(labels = rev(new_labels), limits = rev)
```

```
p<-grid.arrange(p1,p2,ncol = 2)
```

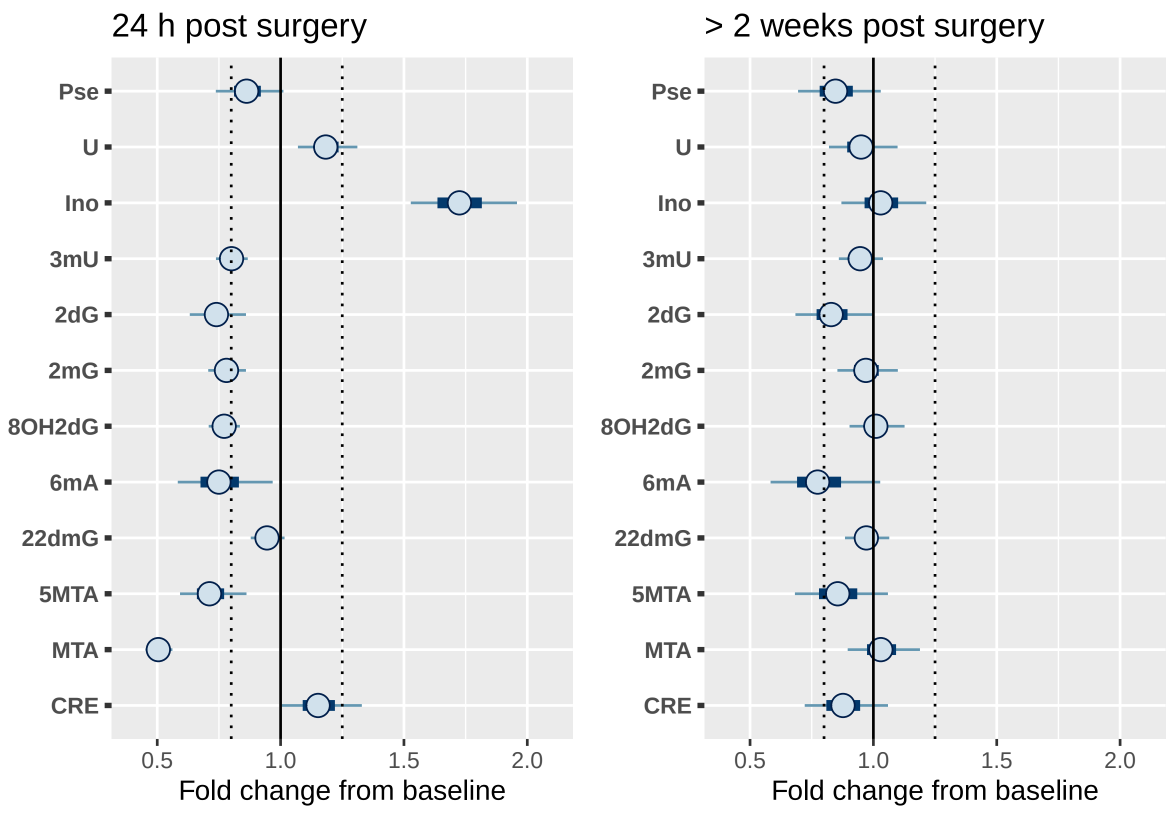


```
ggsave("Manuscript/Effects.png", plot=p,  width = 15*1.4, height = 15, units = "cm", dpi=300)
```

There are some large effect at $TimePoint = 2$ that disappear at $TimePoints3$.

Hypothesis testing

The probability of the 0.8-fold decrease and 1.25-fold increase for each MET and TimePoint is:

```
ans1<-hypothesis(fit, c("abs(TimePoint2)>0.223",
                        "abs(TimePoint2:METU)>0.223",
                        "abs(TimePoint2:METIno)>0.223",
                        "abs(TimePoint2:METX3mU)>0.223",
                        "abs(TimePoint2:METX2dG)>0.223",
                        "abs(TimePoint2:METX2mG)>0.223",
                        "abs(TimePoint2:METX8OH2dG)>0.223",
                        "abs(TimePoint2:METX22dmG)>0.223",
                        "abs(TimePoint2:METX5MTA)>0.223",
                        "abs(TimePoint2:METMTA)>0.223",
                        "abs(TimePoint2:METCRE)>0.223"
                        ))
ans2<-hypothesis(fit, c("abs(TimePoint3)>0.223",
                        "abs(TimePoint3:METU)>0.223",
```

```
                        "abs(TimePoint3:METIno)>0.223",
                        "abs(TimePoint3:METX3mU)>0.223",
                        "abs(TimePoint3:METX2dG)>0.223",
                        "abs(TimePoint3:METX2mG)>0.223",
                        "abs(TimePoint3:METX8OH2dG)>0.223",
                        "abs(TimePoint3:METX22dmG)>0.223",
                        "abs(TimePoint3:METX5MTA)>0.223",
                        "abs(TimePoint3:METMTA)>0.223",
                        "abs(TimePoint3:METCRE)>0.223"
                        ))


ans1$hypothesis$Hypothesis=c("abs(T2:PSU)>0.223",
                        "abs(T2:U)>0.223",
                        "abs(T2:Ino)>0.223",
                        "abs(T2:X3mU)>0.223",
                        "abs(T2:X2dG)>0.223",
                        "abs(T2:X2mG)>0.223",
                        "abs(T2:X8OH2dG)>0.223",
                        "abs(T2:X22dmG)>0.223",
                        "abs(T2:X5MTA)>0.223",
                        "abs(T2:MTA)>0.223",
                        "abs(T2:CRE)>0.223")
ans2$hypothesis$Hypothesis=c("abs(T3:PSU)>0.223",
                        "abs(T3:U)>0.223",
                        "abs(T3:Ino)>0.223",
                        "abs(T3:X3mU)>0.223",
                        "abs(T3:X2dG)>0.223",
                        "abs(T3:X2mG)>0.223",
                        "abs(T3:X8OH2dG)>0.223",
                        "abs(T3:X22dmG)>0.223",
                        "abs(T3:X5MTA)>0.223",
                        "abs(T3:MTA)>0.223",
                        "abs(T3:CRE)>0.223")
ans1
```

```
Hypothesis Tests for class b:
              Hypothesis Estimate Est.Error CI.Lower CI.Upper Evid.Ratio
1       abs(T2:PSU)>0.223    -0.07      0.09    -0.20     0.08       0.26
2         abs(T2:U)>0.223    -0.05      0.06    -0.16     0.05       0.23
3       abs(T2:Ino)>0.223     0.32      0.08     0.20     0.45    3999.00
4      abs(T2:X3mU)>0.223     0.00      0.05    -0.08     0.08       0.95
5      abs(T2:X2dG)>0.223     0.08      0.09    -0.07     0.24       4.25
6      abs(T2:X2mG)>0.223     0.03      0.06    -0.07     0.12       2.00
7   abs(T2:X8OH2dG)>0.223     0.04      0.05    -0.04     0.12       3.26
8    abs(T2:X22dmG)>0.223    -0.16      0.04    -0.22    -0.09       0.00
9     abs(T2:X5MTA)>0.223     0.11      0.11    -0.07     0.30       5.11
10      abs(T2:MTA)>0.223     0.46      0.07     0.35     0.57        Inf
11      abs(T2:CRE)>0.223    -0.08      0.08    -0.20     0.06       0.21
   Post.Prob Star
1       0.21
2       0.19
3       1.00    *
```

```
4       0.49
5       0.81
6       0.67
7       0.76
8       0.00
9       0.84
10      1.00    *
11      0.17
---
'CI': 90%-CI for one-sided and 95%-CI for two-sided hypotheses.
'*': For one-sided hypotheses, the posterior probability exceeds 95%;
for two-sided hypotheses, the value tested against lies outside the 95%-CI.
Posterior probabilities of point hypotheses assume equal prior probabilities.
```

```
ans2
```

```
Hypothesis Tests for class b:
              Hypothesis Estimate Est.Error CI.Lower CI.Upper Evid.Ratio
1      abs(T3:PSU)>0.223    -0.05      0.11    -0.20     0.14       0.46
2        abs(T3:U)>0.223    -0.14      0.06    -0.22    -0.02       0.03
3      abs(T3:Ino)>0.223    -0.14      0.06    -0.22    -0.02       0.03
4     abs(T3:X3mU)>0.223    -0.16      0.05    -0.22    -0.07       0.00
5     abs(T3:X2dG)>0.223    -0.03      0.11    -0.20     0.16       0.60
6     abs(T3:X2mG)>0.223    -0.16      0.05    -0.22    -0.06       0.01
7  abs(T3:X8OH2dG)>0.223    -0.17      0.04    -0.22    -0.09       0.00
8   abs(T3:X22dmG)>0.223    -0.17      0.04    -0.22    -0.10       0.00
9    abs(T3:X5MTA)>0.223    -0.05      0.11    -0.21     0.16       0.44
10     abs(T3:MTA)>0.223    -0.15      0.06    -0.22    -0.05       0.02
11     abs(T3:CRE)>0.223    -0.08      0.10    -0.21     0.10       0.28
   Post.Prob Star
1       0.32
2       0.03
3       0.03
4       0.00
5       0.37
6       0.01
7       0.00
8       0.00
9       0.31
10      0.02
11      0.22
---
'CI': 90%-CI for one-sided and 95%-CI for two-sided hypotheses.
'*': For one-sided hypotheses, the posterior probability exceeds 95%;
for two-sided hypotheses, the value tested against lies outside the 95%-CI.
Posterior probabilities of point hypotheses assume equal prior probabilities.
```

```
#plot(ans1)
```

There is a clear evidence the concentrations of Ino has increased and MTA has decreased 24 post surgery.

Variation in duresis

```
fit %>%
    spread_draws(r_Sample[condition,]) %>%
    median_qi(condition_mean = r_Sample, .width = c(.95, 0.66))%>%
    ggplot(aes(y = reorder(condition,condition_mean), x = condition_mean, xmin = .lower, xmax = .upper))
    geom_pointinterval() +
    theme(axis.text = element_text(size = 5))+
    xlab("Change in diuresis (log scale)") +
    ylab("Sample")
```

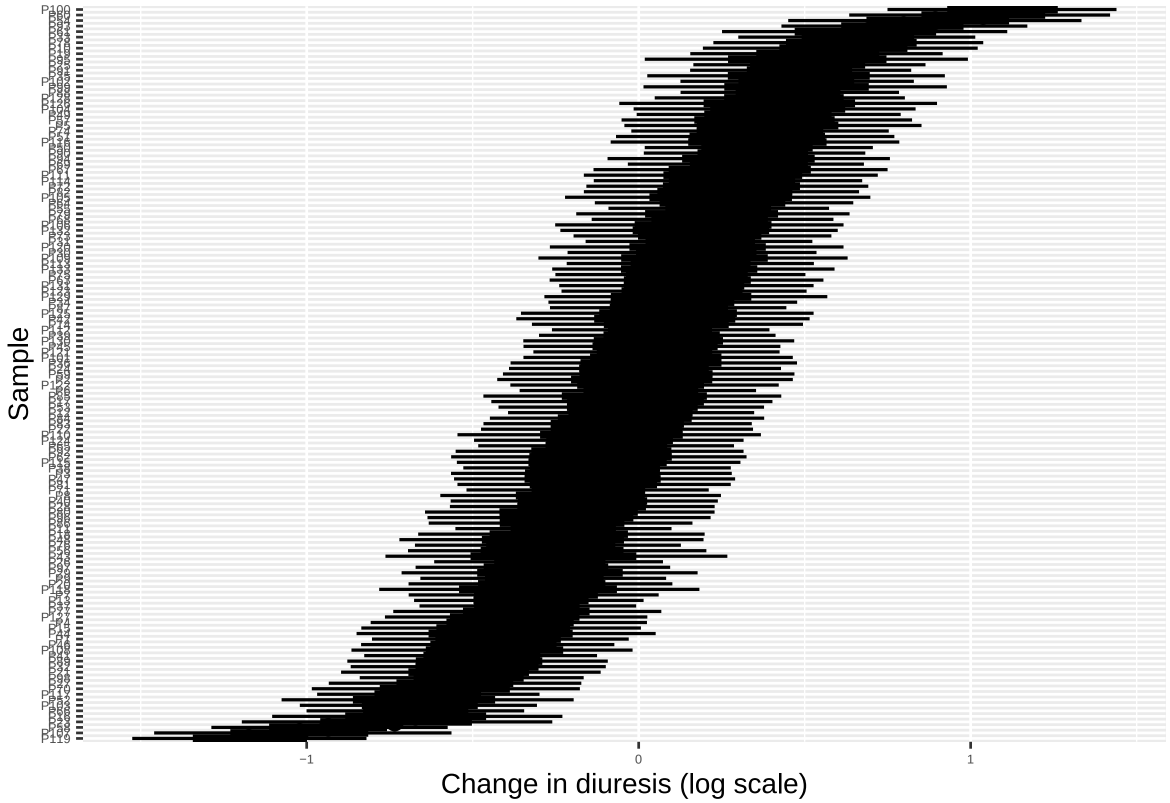

Conclusions

- The lack of control group limits the possibility to answer whether there is an effect of cancer removal on urine concentration of nucleosides and deoxynucleosides. It is possible that the observed differences are related to the surgical procedure itself.
- MTA and Ino have the largest negative and positive effect at TimePoint2 (relative to Timepoint1). The effects are however temporary and return to pretreatment levels.

Session info

```r
sessionInfo()
```

```
R version 4.1.3 (2022-03-10)
Platform: x86_64-w64-mingw32/x64 (64-bit)
Running under: Windows 10 x64 (build 22621)

Matrix products: default

locale:
[1] LC_COLLATE=Polish_Poland.1250  LC_CTYPE=Polish_Poland.1250
[3] LC_MONETARY=Polish_Poland.1250 LC_NUMERIC=C
[5] LC_TIME=Polish_Poland.1250

attached base packages:
[1] stats     graphics  grDevices utils     datasets  methods   base

other attached packages:
[1] tidyr_1.3.0     tidybayes_3.0.2 knitr_1.40      GGally_2.1.2
[5] brms_2.18.0     Rcpp_1.0.9      gridExtra_2.3   ggplot2_3.4.2
[9] dplyr_1.1.2

loaded via a namespace (and not attached):
 [1] nlme_3.1-155         matrixStats_0.62.0   xts_0.12.1
 [4] RColorBrewer_1.1-3   threejs_0.3.3        rstan_2.21.7
 [7] tensorA_0.36.2       tools_4.1.3          backports_1.4.1
[10] utf8_1.2.2           R6_2.5.1             DT_0.25
[13] ggdist_3.2.0         colorspace_2.0-3     withr_2.5.0
[16] tidyselect_1.2.0     prettyunits_1.1.1    processx_3.7.0
[19] Brobdingnag_1.2-7    compiler_4.1.3       textshaping_0.3.6
[22] cli_3.4.0            arrayhelpers_1.1-0   shinyjs_2.1.0
[25] labeling_0.4.2       colourpicker_1.1.1   posterior_1.3.1
[28] scales_1.2.1         dygraphs_1.1.1.6     checkmate_2.1.0
[31] mvtnorm_1.1-3        ggridges_0.5.3       callr_3.7.2
[34] systemfonts_1.0.4    stringr_1.5.0        digest_0.6.29
[37] StanHeaders_2.21.0-7 rmarkdown_2.16       base64enc_0.1-3
[40] pkgconfig_2.0.3      htmltools_0.5.3      fastmap_1.1.0
[43] htmlwidgets_1.5.4    rlang_1.1.0          rstudioapi_0.14
[46] shiny_1.7.2          svUnit_1.0.6         farver_2.1.1
[49] generics_0.1.3       zoo_1.8-11           crosstalk_1.2.0
[52] gtools_3.9.3         distributional_0.3.1 inline_0.3.19
[55] magrittr_2.0.3       loo_2.5.1            bayesplot_1.9.0
[58] Matrix_1.4-0         munsell_0.5.0        fansi_1.0.3
[61] abind_1.4-5          lifecycle_1.0.3      stringi_1.7.8
[64] yaml_2.3.5           pkgbuild_1.3.1       plyr_1.8.7
[67] grid_4.1.3           parallel_4.1.3       promises_1.2.0.1
[70] crayon_1.5.1         miniUI_0.1.1.1       lattice_0.20-45
[73] ps_1.7.1             pillar_1.9.0         igraph_1.3.5
[76] markdown_1.1         shinystan_2.6.0      reshape2_1.4.4
[79] codetools_0.2-18     stats4_4.1.3         rstantools_2.2.0
[82] glue_1.6.2           evaluate_0.16        RcppParallel_5.1.5
[85] vctrs_0.6.2          httpuv_1.6.6         purrr_1.0.1
[88] gtable_0.3.1         reshape_0.8.9        xfun_0.32
[91] mime_0.12            xtable_1.8-4         coda_0.19-4
```

```
[94] later_1.3.0          ragg_1.2.5          tibble_3.2.1
[97] shinythemes_1.2.0    ellipsis_0.3.2      bridgesampling_1.1-2
```
